# Hsp90 Inhibitors Are Efficacious against Kaposi Sarcoma by Enhancing the Degradation of the Essential Viral Gene LANA, of the Viral Co-Receptor EphA2 as well as Other Client Proteins

**DOI:** 10.1371/journal.ppat.1003048

**Published:** 2012-11-29

**Authors:** Wuguo Chen, Sang-Hoon Sin, Kwun Wah Wen, Blossom Damania, Dirk P. Dittmer

**Affiliations:** Department of Microbiology and Immunology, Program in Global Oncology, Lineberger Comprehensive Cancer Center, Center for AIDS Research, University of North Carolina at Chapel Hill, Chapel Hill, North Carolina, United States of America; University of Southern California Keck School of Medicine, United States of America

## Abstract

Heat-shock protein 90 (Hsp90) inhibitors exhibit activity against human cancers. We evaluated a series of new, oral bioavailable, chemically diverse Hsp90 inhibitors (PU-H71, AUY922, BIIB021, NVP-BEP800) against Kaposi sarcoma (KS). All Hsp90 inhibitors exhibited nanomolar EC_50_ in culture and AUY922 reduced tumor burden in a xenograft model of KS. KS is associated with KS-associated herpesvirus (KSHV). We identified the viral latency associated nuclear antigen (LANA) as a novel client protein of Hsp90 and demonstrate that the Hsp90 inhibitors diminish the level of LANA through proteasomal degradation. These Hsp90 inhibitors also downregulated EphA2 and ephrin-B2 protein levels. LANA is essential for viral maintenance and EphA2 has recently been shown to facilitate KSHV infection; which in turn feeds latent persistence. Further, both molecules are required for KS tumor formation and both were downregulated in response to Hsp90 inhibitors. This provides a rationale for clinical testing of Hsp90 inhibitors in KSHV-associated cancers and in the eradication of latent KSHV reservoirs.

## Author Summary

Heat shock proteins, such as Hsp90, aid the folding of proteins. They seem to be essential to sustain the growth of cancer cells. Hsp90 inhibitors are in clinical trials for many cancers but with mixed results, presumably since these proteins have many clients. The mechanism for drug efficacy and tumor-type variation in responses is not understood. Here we show that in the case of Kaposi sarcoma and primary effusion lymphoma, which are cancers caused by Kaposi sarcoma associated herpesvirus (KSHV/HHV8) an essential viral protein, LANA, binds to Hsp90 and is a client of Hsp90. Different small molecule Hsp90 inhibitors reduce the expression of LANA. At the same time they reduce the expression of the newly discovered co-receptor of KSHV ephA2, of Akt, cdc2 and ephrin-B2. Since LANA is required to maintain the virus latent in all tumor cells, a process, which is periodically aided by de novo infection, these inhibitors interfere with essential components of viral pathogenesis and in vivo tumor growth.

## Introduction

Heat shock protein 90 (Hsp90) is a conserved molecular chaperone that facilitates the maturation of a wide range of proteins and assists in the correct folding and productive assembly of cellular proteins and multimeric protein complexes in normally growing cells [1], [2]. Hsp90 also has important roles in maintaining the transformed phenotype of cancer cells. Overexpression of Hsp90 has been detected in a variety of cancers [3], [4], [5]. Hsp90 is required for proper folding of its “client proteins” many of which are effectors of key signal transduction pathways controlling cell growth, differentiation, the DNA-damage response, and cell survival [6]. Cancer cells are critically addicted to the Hsp90 chaperone machinery whose activity protects an array of mutated and overexpressed oncoproteins, and other cellular client proteins from misfolding and degradation [7], [8].

Hsp90 is an emerging therapeutic target for cancer [8], [9], [10]. The newer class of Hsp90 inhibitors bind to the ATP-binding motif of Hsp90 and inhibit its protein chaperoning activity, resulting in misfolding, subsequent degradation of cellular client proteins, and ultimately tumor cell death [4], [7], [11], [12]. Hsp90 inhibitors are selective for tumor cells because the chaperoning function of Hsp90 is required for most tumor cells. Even though the new inhibitors are highly selective for Hsp90, Hsp90 has many client proteins, each of which can contribute to the transformed phenotype. For instance, Hsp90 is involved in NFκB activation by IKK [13] in normal and lymphoma cells, including in the Kaposi sarcoma-associated herpesvirus (KSHV) driven lymphoma cell lines [14], [15]. Additionally, soluble extracellular Hsp90 has been implicated in supporting de novo infection by KSHV [16].

We focused our attention on (i) ephrins and ephrin receptors because of their connection to Kaposi sarcoma (KS) and Kaposi sarcoma associated herpesvirus (KSHV) infection and (ii) on the KSHV latency associated nuclear antigen (LANA), which is essential for maintaining the KSHV virus and thereby the transformed phenotype [17]. Kaposi sarcoma (KS) is an endothelial cell lineage cancer; in fact, KS is one of the most vascular human cancers.

Ephrin interactions can trigger a wide array of cellular responses, including cell adhesion, boundary formation and repulsion [18]. Ephrin-A1 for instance was discovered as a TNF-inducible protein in HUVEC cells. Ephrins are membrane bound by glycosylphosphatidylinositol (GPI) anchor in case of ephrin-A1 to A5 and a transmembrane domain in case of ephrin-B1 to B5. They form receptor ligand pairs with ephrin receptors.

Ephrin-B2 plays critical roles in vessel maturation. It is expressed on endothelial cells, arterial angioblasts and perivascular mesenchymal cells. Ephrin-B2 is expressed at substantial levels in KS, KS cell lines, transformed lymphatic endothelial cells (LEC/HHV-8), and in KS tissue [19], [20]. The continued presence of KSHV and expression of viral proteins are essential for the development of KS, and KSHV can reprogram primary endothelial cells to extend their life-span and to acquire features of transformation [21], [22], [23], [24], [25], [26], [27]. Ephrin-B2 signals through the EphB4 receptor.

EphA2 is a receptor for ephrin-A1 [28]. Ephrin receptors are receptor tyrosine kinases. EphA2 has previously been identified as an Hsp90 client protein [29], [30]. It is overexpressed in a large number of human malignancies and supports tumor angiogenesis [29], [30]. Targeting the ephrin-ephrin receptor interactions by antibodies, siRNA, or soluble ligands (e.g soluble EphB4, the receptor for ephrin-B2, fused to albumin [31]) disrupts endothelial cell function and tumor vasculature [32], [33]. The first clinical studies targeting ephrin interactions are currently in design. This establishes ephrins as key regulators of tumor angiogenesis and endothelial cell growth.

EphA2 also has a newly identified direct role in KSHV infection of endothelial cells. EphA2 has been established as a co-receptor of KSHV, binding to the viral gH and gL proteins [34], and as a mediator of KSHV-induced signaling [35]. Because initial infection of endothelial cells with KSHV is a prerequisite for them to eventually become KS tumor cells, and because periodic re-infection seems to contribute to viral maintenance and tumor progression, any drug that interferes with latency (via targeting LANA) and lowers re-infection (via targeting ephrin) would significantly impact KS pathogenesis.

Like other herpesviruses, KSHV exhibits two distinct phases in its life cycle, latent and lytic replication. During latent infection, only a small subset of viral proteins is expressed in the KS tumor cells chiefly the latency-associated nuclear antigen (LANA) [36], [37]. LANA is necessary and sufficient for episome persistence of KSHV [38]; it is required for tumor cell survival [17]. LANA can interact with a multitude of partners [39], [40], [41], [42], [43], including tumor suppressor proteins, leading to the inhibition of apoptosis and dysregulation of the cell cycle [44], [45], [46]. These activities contribute to tumor cell survival and cell proliferation. Thus viral latent proteins constitute a highly specific target for KS cancer therapy.

In the present work, we discovered that Hsp90 is an essential regulator of LANA, ephA2 and ephrin-B2. Multiple, highly specific, chemically distinct and oral bioavailable Hsp90 inhibitors were used to treat PEL and KS tumor lines. This accelerated the degradation of LANA through the proteasomal pathway and downregulated ephrin B2 levels. The result was growth inhibition in culture at nanomolar concentrations, and tumor retardation in a xenograft model of KS.

## Materials and Methods

### Cell culture, antibodies and drugs

BJAB, BJAB-LANA derivatives and the KSHV positive PEL cell lines BC-3, BCP-1, BCBL-1, and BC-1 were cultured in RPMI 1640 medium (Cellgro) containing 2 mM L-glutamine, 10% fetal bovine serum, penicillin G (100 U/ml) and streptomycin sulfate (100 µg/ml) and supplemented with 0.05 mM 2-mercaptoethanol (Sigma), 0.075% sodium bicarbonate (Life Technologies), and 1 U/ml human interleukin-6 (IL-6) (Roche). SLK, SLK-KSHV, L1T2, TIVE-L1, KS-IMM and HeLa cell lines were cultured in DMEM (Cellgro) supplemented with 100 µg/ml streptomycin, 100 U/ml penicillin G (Life Technologies, Carlsbad, CA, USA), and 10% FBS at 37°C in 5% CO_2_. SLK-KSHV cells (gift of Dr. Don Ganem [47]) were maintained with additional puromycin (1 µg/ml, Invitrogen).

Rat anti-LANA monoclonal LN53 was purchased from Advanced Biotechnology Inc., anti-LANA polyclonal rabbit antiserum YT041 was raised again the LANA repeat region [48], and mouse anti-LANA (13B10) was from Leica Biosystems Newcastle Ltd. Rabbit cleaved PARP (Asp214, D64E10), Cleaved caspase-3 (Asp175, 5A1E), rabbit Anti-Akt and phospho-Akt (Ser473, D9E) were purchased from Cell Signaling. Mouse Anti-β-Actin, mouse anti-hemagglutinin (anti-HA, clone HA-7) and mouse anti-FLAG (clone M2) antibodies were purchased from Sigma Inc. Anti-ephrin B2 antibody was purchased from R&D Systems (catalog number: AF496, Minneapolis, MN). Mouse anti-Cdc-2 p34 (17, sc-54) and rabbit anti-EphA2 (C-20, sc-924) antibodies were from Santa Cruz. Mouse anti-Hsp90β (SPA-842) and anti-Hsp90 (Ab1429) antibodies were purchased from Stressgen and Abcam Inc, respectively. Hsp90 inhibitor 17-DMAG was from Invivogen Inc.; and PU-H71 from Sigma Inc.. BIIB021, NVP-AUY922 and NVP-BEP800 were purchased from Selleck.

### MS/MS analysis for LANA complexes

FLAG-tagged LANA (kindly provided by Dr. Ken Kaye [42]) was cloned into pcDNA3 to yield pDD1946 (aa1–1162). Flag-HA-double tagged central-repeat deleted (1-329+928-1162aa) expression construct was cloned into pcDNA3 to yield pDD1945 as follows: The central repeat LANA mutant pMF-24 was kindly provided by Dr. Diane Hayward [45]. Both tagged LANA mutants were transfected into BJAB cells with lipofectamin 2000 (Invitrogen). Stable BJAB cells were selected with G-418 (1 mg/ml, Gibco). Approximately 5×10^9^ cells were harvested after large-scale culture. Nuclear extraction of BJAB (FLAG-LANA) cells was performed as previously described, followed by two chromatographic columns of Sepharose 6B and Heparin FF [48]. Isolated samples from chromatographic columns were further purified by another two-step immunoaffinity method, first by incubation with 50 µl EZ view anti-FLAG M2 affinity resin (Sigma) in TBS (10 mM Tris-HCl, 100 mM NaCl [pH 8.0]) overnight at 4°C, then the FLAG-tagged protein was eluted by 200 µl of 150 µg/ml 3×FLAG peptide (Sigma), washed three times and diluted with cold RIPA buffer (150 mM 150 mM NaCl, 0.5% NP-40, 50 mM Tris-HCl [pH 8.0], 1 mM EDTA, 0.5 mM DTT, 0.5 mM PMSF, 0.5% cocktail protein inhibitor (Sigma)). Finally, rat anti-LANA (clone LN53) or mouse anti-HA (clone HA-7) was used for further purification of LANA complexes, rat IgG was used for control (Sigma). Purified proteins were resolved by 8 to 16% gradient SDS-PAGE and stained with colloidal blue (Invitrogen). Visible bands were cut and further subjected to mass spectrometry at the University of North Carolina—Chapel Hill core facility.

### Immunoprecipitation and Western blotting

A series of full length or FLAG-LANA mutant expressing plasmids (pDD1928 (aa1–329), pDD1931 (aa930–1162) and pDD775) were obtained from Dr. Diane Hayward [45], [49]. These together with HA-Hsp90 [50] were co-transfected separately into HeLa cells and harvested after 48 hours. Mab mouse anti-HA and anti-FLAG were used in immunoprecipitation assay as previously described [48]; mouse IgG was used as control. Samples were washed with cold RIPA buffer (150 mM 150 mM NaCl, 0.5% NP-40, 50 mM Tris-HCl [pH 8.0], 1 mM EDTA, 0.5 mM DTT, 0.5 mM PMSF, 0.5% cocktail protein inhibitor), followed by SDS-PAGE analysis and transferred into Hybond P membranes (Amersham), secondary antibodies conjugated with horseradish peroxidase (HRP) (1∶1000 anti-mouse IgG (Vector Labs), 1∶1000 anti-rabbit IgG (Vector labs)) were incubated and exposed to X-film (Genesee).

### Immunofluorescence assay

TIVE-L1 cells were cultured overnight on glass coverslips in 6-well plates. After fixation with 3% paraformaldehyde for 20 min and permeabilization with 0.2% Triton X-100 for 15 min, cells were incubated in blocking buffer (TBS+10% horse serum) following by rabbit anti-LANA YT041 (diluted 1∶500) or mouse anti-Hsp90 (diluted 1∶500). Slides were then incubated with appropriate secondary antibody anti-rabbit Texas red conjugated or anti-mouse FITC-conjugated (Vector laboratory) and counterstained with DAPI. Images were obtained using a Leica model DM4000B microscope, with 100-fold magnification; software, SimplePCI version 6.2).

### Immunohistochemistry

Solid tumors were fixed in 10% neutral buffered formalin for 2 days, and paraffin-embedded. Following procedures previously described [51], slides were first deparaffinized using Histochoice Clearing Agent (Sigma) and then rehydrated. Endogenous peroxidase activity was quenched with 3% H_2_O_2_ in 10% methanol, then sections were blocked in solution B (10% horse serum [Vector laboratory], 5% BSA and 0.3% Triton X-100 in PBS) for 1 hour at RT, followed by incubation overnight at 4°C with primary antibodies: phospho-Akt (S473, 1∶100), LANA (ABI, 1∶200), and ephrin B2 (1∶100); solution B was used as negative control. After washing in PBS, sections were incubated with appropriate biotinylated secondary antibodies followed by Avidin DH (Vectastain ABC kit, Vector laboratory) administration, after which sections were stained with Vector NovaRed substrate (Vector laboratory). Slides were counterstained with hematoxylin (Sigma), dehydrated using graded alcohols, cleared in xylene and mounted in Permount (Sigma). Images were observed using Leica DM LA histology microscope equipped with a 10×/0.25 numerical aperture or a 40×/0.75 numerical aperture N plan objective and a Leica DPC 480 camera.

### Knockout of Hsp90 with shRNA lentivirus

Two pLKO.1 lentiviral vectors (TRCN000008749, Target sequence: 5′-CGCATGATCAAGCTAGGTCTA and TRCN000008750, Target sequence: 5′-CCAACTCATGTCCCTCATCAT) targeted for Hsp90 (NM_007355) were obtained from Open Biosystems/Thermo Inc. The reconstructed lentiviruses were produced by the Lenti-shRNA Core Facility of University of North Carolina. BC-1 and BCBl-1 cells (5×10^5^) were grown in six-well plates, infected with lentiviruses separately by adding polybrene at a final concentration of 10 µg/ml and incubated at 37°C for 6 hrs. After infection, BC-1 and BCBL-1 cell medium was replaced with fresh RPMI 1640 supplemented with 10% fetal bovine serum. On the second day, puromycin (5 µg/ml) was added to the medium. Finally, all the cells were harvested after infection for four days. Empty lentivirus (shRNA) or untreated BC-3 cells were used as controls.

### IC50 assay

The real-time growth of adherent cells was monitored by xCELLigence system (Roche Diagnostics, Indiana, IN). 2500 cells of L1T2, KS-IMM, SLK and SLK-KSHV were seeded on specialized microplates that contain microelectronic sensor arrays at the bottom of the wells. Different concentrations of Hsp90 inhibitors (17-DMAG, PU-H71, NVP-AUY922, BIIB021 and NVP-BEP800) or vehicle were added to the plate after 20 hours of cellular growth. Two-fold serial dilutions of 17-DMAG, PU-H71, BIIB021, NVP-BEP800 (0, 10, 20, 40, 80, 160, 320 and 640 nM) or NVP-AUY922 (0, 2, 4, 8, 16, 32 and 64 nM) were used for analyses. IC50 was determined at the time of 72 or 96 hours dependent on cell type after growing for up to 120 hours. Each experiment was repeated twice. Live cells that adhere to the bottom of the well result in higher impedance (Cell Index), and dying cells lose contact thereby lowering the Cell Index. Effect of drug treatment on KS cells was determined by monitoring the electronic impedance every 30 min over a period of 120 hours. Growth curves and IC50 plots were generated using RTCA Software v1.2 (ACEA Bio. Inc.).

### Colony formation assay

400 L1T2 cells were seeded after counting in 10 cm dish with growth media (as above) supplemented either with vehicle or drugs at serially diluted concentrations (0, 10, 20, 40 and 80 nM for 17-DMAG, PU-H71 and BIB021; 0, 20, 40, 80 and 160 nM for NVP-BEP800; 0, 1, 2, 4 and 8 nM for AUY922). Growth of colony formation was monitored over a period of 2-weeks. At the end of the assay, colonies were stained with Magic Blue Stain (3 g crystal violet and 0.92 g of Ammonium oxalate in 20% ethanol) and assessed visually by counting the number of colonies formed. Each experiment was repeated three times.

### Real-time quantitative PCR

0.5 µM 17-DMAG was added into PEL cells (BC-3, BCP-1, BCBL-1 and BC-1), and cells were harvested after 0, 12 and 24 hours. Total RNA was isolated by TRI REAGENT (Sigma; Saint Louis, MO) and purified by Oligotex mRNA purification system (Qiagen, CA) according to supplier's protocol. Reverse transcription was performed with 0.5 µg random hexanucleotide primers (Amersham Pharmacia Biotech). Real-time qPCR was used to detect the viral genome as previously described [52]. The values were relative to the housekeeping gene GAPDH. Primers were used as listed: LANA forward (5′-3′): CGAGAGGAAGTTGTAGGAAACG, LANA reverse (5′-3′): CTTCCAGGTATAGGCAAGGTG; Rta forward (5′-3′): TGTAATGTCAGCGTCCACTC, Rta reverse (5′-3′): ATTGCTGGGCCACTATAACC; GAPDH forward (5′-3′): ACATCGCTCAGACACCATG, GAPDH reverse (5′-3′): TGTAGTTGAGGTCAATGAAGGG.

### Flow cytometry assay

For cell cycle analysis, cells fixed in 70% ethanol were resuspended in phosphate-buffered saline with 20 µg/ml propidium iodide, 200 µg/ml RNase A and 0.1% Triton X-100. For apoptosis analysis, cells were stained with FITC conjugated anti-Annexin V antibody. Flow cytometry analysis was performed by using CyAn (Beckman-Coulter, Fullerton, CA). Further analysis was conducted with FlowJo (version 7.6.5; Tree Star, Inc., Ashland, OR) and R version 2.15.1 (2012-06-22).

### Animal studies

1×10^5^ L1T2 cells were counted after washing with PBS once, diluted into 100 µl PBS and mixed with 100 µl growth factor-depleted Matrigel (BD Biosciences, Bedford, MA). 1×10^5^ cells were injected sub-cutaneous into the flank of C.B.-17 SCID mice (Jackson Laboratory, Bar Harbor, MN) following our previously validated procedures [53]. Two groups were used for experiment and control; each group had 6 mice. The mice were observed every one or two days for the presence of palpable tumors. Three days post-injection, a single dose of 50 mg/kg AUY922 or vehicle was injected intra-peritoneal as previously described [54]. Tumor diameters were determined by caliper measurements. Tumor volume was calculated as V = a * b * c, where a, b, and c are the three diameters (length, breadth and width) of the tumor. The tumors were excised from the site of injection and fixed in formalin (Fisher Diagnostics, Middletown, VA).

## Results

### Hsp90 interacts with KSHV LANA

**Figure 1:**
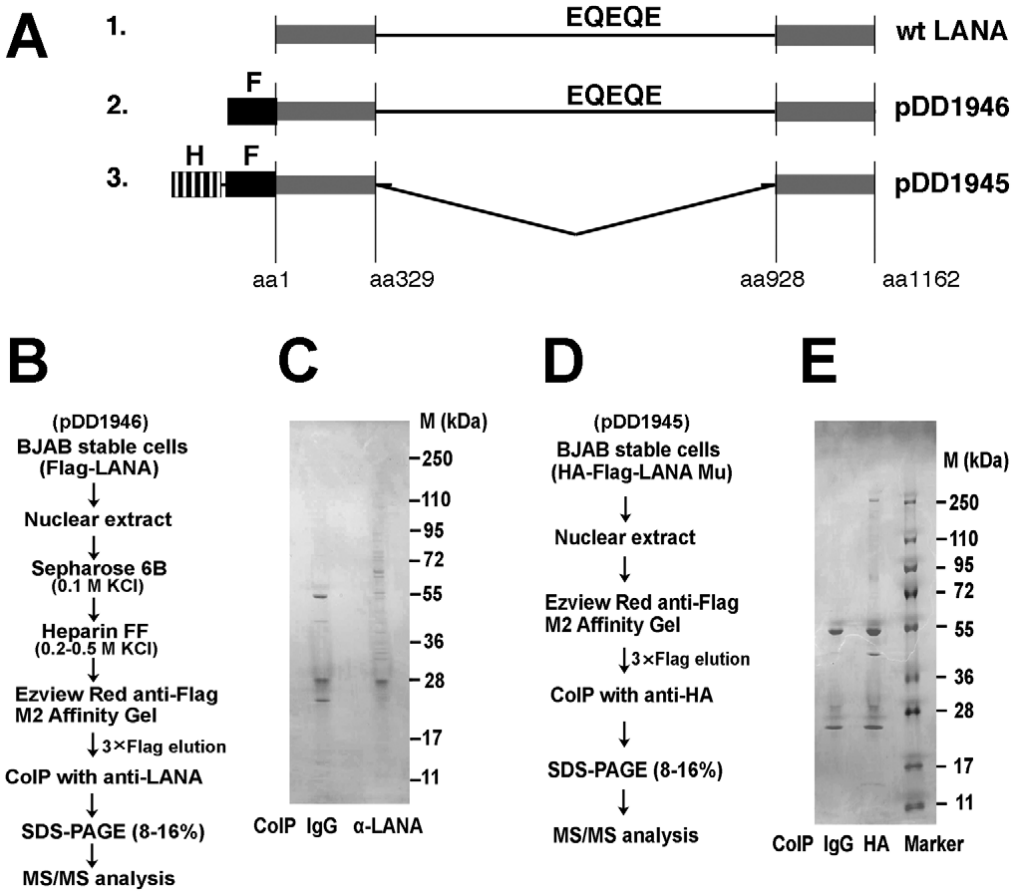
Purification and identification of LANA binding proteins. (A) Outline of biochemical purification baits all of which pulled down Hsp90; (1.) wild-type, unmodified LANA; (2.) as used to derive partners listed in table 1; (3.) as used to derive partners listed in table 2. The abbreviations denote epitopes: F for DYKDDDDK, HA for YPYDVPDYA and EQEQE which is repeated within the LANA central domain. (B) Schematic procedure for purification of LANA complexes using bait 2. Samples were eluted from columns of Sepharose 6B and Heparin FF for purification separately, followed by immunoaffinity purification with mouse anti-Flag M2 affinity gel and immunoprecipitated using anti-LANA mab. (C) SDS-PAGE analysis was performed by 8–16% gradient gel and stained with colloidal blue. (D) Schematic procedure for purification of LANA mutant complexes using bait 3. (E) SDS-PAGE analysis was performed by 8–16% gradient gel and stained with colloidal blue. Co-IP, immunoprecipitation; M, molecular mass (in kDa).

LANA is essential for maintaining latent KSHV, which is a prerequisite for PEL and KS tumorigenesis. Thus, it is of continued interest to identify cellular binding partners of LANA. We previously purified authentic LANA complexes from the BC-3 PEL cell line [40]. In the context of PEL (and KS) most of the LANA is tethered to the viral episome. To identify LANA binding partners that are important in protein maturation and in functions of LANA that are not tightly linked to DNA binding we stably expressed full length FLAG-tagged LANA or a mutant in KSHV-negative BJAB cells (**Figure 1A**). Then we used two-step chromatographic isolation (Sepharose 6B and Heparin FF column), followed by sequential immunoaffinity purification (Tag-TAP) with two different monoclonal antibodies; mouse anti-FLAG against the N-terminal epitope tag and rat anti-LANA (against the EQEQE repeat epitope) against the central repeat region (outlined in **Figure 1B**). We previously found that heparin FF bound intact LANA complexes [48] consistent with its established use as initial step in many of the early transcription factor isolation studies. LANA binding proteins were resolved by 8–16% gradient SDS-PAGE (**Figure 1C**) and subjected to MS/MS. We identified heat shock protein Hsp90-beta (NP_0310381). We also found several other heat shock proteins such as HSPA9 protein (AAH30634), and heat shock cognate 71 kDa protein isoform1 (NP_006588) (**Table 1**). This corroborates our prior work, where we co-purified HSPs as one of many binding partners of authentic full length LANA in PEL [40]. To confirm our experiments and because of potential non-specific interactions with the central repeat region we generated a stable BJAB cell line expressing a mutant LANA protein, which had a deletion of the central repeat region, and which was engineered to have both a FLAG and HA tag at the N-terminus (**Figure 1A**). Again we performed Tag-TAP purification on nuclear extracts (**Figure 1D**), resolved individually associated proteins on SDS-PAGE (**Figure 1E**) and identified visible bands by MS/MS. The result confirmed the association with Hsp family members (**Table 2**). These three independent biochemical purifications using different antibodies and different “bait” constructs demonstrate that LANA is associated with cellular heat shock proteins, and that this interaction occurs independently of other viral proteins or viral DNA.

**Table 1:** Result of MS/MS for Flag-LANA associated proteins (<1,000 aa).

*ID*	*Name*	*Size*	*MW (Da)*	*MS*	*Score*	*comment*
CAA46472	HNR hnRNP U protein	806	88890.2	15	106	TR
NP_031381	HSP 90-beta	724	83212.1	31	714	HSP
NP_031381	HSP 90-beta	724	83212.1	19	190	HSP
AAQ88940	disulfide isomerase	747	86071.9	19	332	.
AAH68458	Ezrin	586	69198.6	28	252	.
AAA52614	HSPA5/GRP78 precursor	653	72071.2	16	294	HSP
AAH30634	heat shock HSPA9 protein	681	73808	29	1110	HSP
NP_006588	heat shock cognate 71 kDa	724	70854.2	26	919	HSP
BAG70196	ATP-dependent RNA hel.	614	69077.7	29	872	.
AAS94255	PIG48/chaperonin	545	60540.3	18	188	HSP
NP_004528	nucleosome assembl.	391	45345.9	10	219	.
EAX09924	chaperonin containing TCP1,	547	59440.4	16	274	HSP
CAG33456	HSPC117	505	55227.9	17	329	.
CAI41893	tubulin, beta	426	47736	22	944	.
CAG33059	HNRPH1	449	49099.3	16	482	TR
NP_817126	actin like protein 6A	387	43208.4	11	223	.
BAD97046	IL enhancer binding factor 2	390	43021.2	15	379	.
AAH08633	actin, beta	368	40978.4	20	945	.
NP_919223	HNR A3	378	39570.6	12	224	TR
NP_112533	HNRA2/B1 isoform B1	353	37406.7	30	929	TR
NP_067676	HNR H3 isoform b	331	35216.4	19	391	TR
NP_002128	HNR A2/B1 isoform A2	341	35983.9	31	1260	TR
NP_002127	HNR A1 isoform a	320	34175.2	17	664	TR
CAA27638	Protein G	480	51837.5	7	299	.
NP_057123	homeobox prox 1	244	28050.7	18	294	.
AAC37629	IgG	113	12356.2	7	204	.

HSP: heat shock protein family.

TR: previously identified as KSHV TR binding [91], [92].

**Table 2:** Result of MS/MS for HA-Flag-LANA-dCR (pMF-24) associated proteins (<1,000 aa).

*ID*	*Name*	*Size*	*MW(Da)*	*MS*	*Score*	*comment*
EAW84989	drebrin 1, isoform CRA_a	687	75526.4	22	410	.
CAA46472	hnRNP U protein	806	88890.2	11	152	TR
Q53GZ6	HSP 70 kDa protein 8 iso 1variant	646	70855.2	23	591	HSP
AAB62657	**LANA (orf73)**	1089	126157.6	13	287	.
AAH30634	heat shock HSPA9 protein	681	73808	26	651	HSP
NP_006588	heat shock cognate 71 kDa	646	70854.2	21	202	HSP
NP_033784	serum albumin precursor	608	68647.7	12	419	.
AAH08237	IgH protein	463	51326.5	10	288	.
AAH08237	IgH protein	463	51326.5	5	682	.
AAH12854	ACTB	360	40194.1	15	179	.
AAH08633	actin, beta	368	40978.4	26	1560	.
AAH08237	IgH protein	463	51326.5	10	160	.
CAA27638	Protein G	480	51837.5	5	304	.
AAH07308	RPS4X protein	263	27242.7	7	66	RIBO
2J4W_L	Chain L	213	23246.1	11	633	.

HSP: heat shock protein family.

TR: previously identified as KSHV LANA-TR binding [91], [92].

RIBO: previously identified as LANA binding in PEL [40].

**Figure 2:**
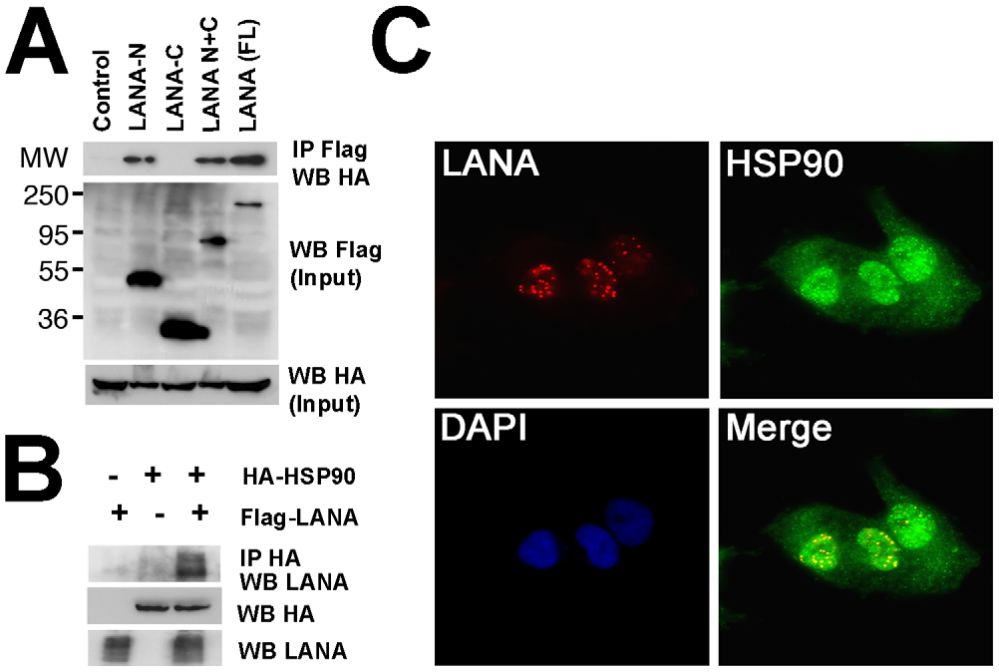
Analysis of interaction between LANA and Hsp90. (A) Interaction between Hsp90 and LANA or respective LANA mutants in HeLa cells. HA-tagged Hsp90 was co-transfected with Flag-tagged full-length LANA (aa1–1162) or indicated mutants: Flag-tagged LANA-N (aa1–329, pDD1928), Flag-tagged LANA-C(aa930–1162,pDD1931), or Flag-tagged LANA N+C (aa1–329 and 928–1162, pDD775). Protein extracts were immunoprecipitated with anti-Flag antibody followed by immunoblotting with anti-HA antibodies, IgG was used as control. Input samples were from cell lysate supernatants. MW markers in kD are indicated on the left. (B) Reverse immunoprecipitation. HA-tagged Hsp90 together with Flag-tagged LANA were co-transfected into HeLa cells, empty pcDNA vector was used for control. Protein extracts were immunoprecipitated with anti-HA antibody and immunoblotted with anti-LANA antibody. Input samples were from cell lysate supernatant. (C) Co-localization analysis of LANA and Hsp90. Immunofluorescence assay was performed after fixation and permeabilization of TIVE-L1 cells, incubated with primary rabbit anti-LANA and mouse anti-Hsp90 antibodies and the secondary anti-rabbit Texas (red) and anti-mouse FITC (green) conjugated antibodies respectively. Nuclear fractions were stained with DAPI, images were observed under fluorescence microscope.

To investigate the interaction between LANA and Hsp90, we used WT FLAG-tagged LANA and FLAG-tagged mutant derivatives, the N-terminal or C-terminal of LANA (or both). After co-transfection of full-length FLAG tagged LANA (or LANA mutants) and HA-tagged-Hsp90 in HeLa cells, immunoprecipitation was performed with anti-FLAG antibody to bait Hsp90 complexes; the complexes separated by SDS-PAGE and associated protein detected with anti-HA antibody. We found that full-length LANA bound to Hsp90, and that the N-terminal of LANA but not the C-terminal interacts with Hsp90 (**Figure 2A**). The reverse immunoprecipitation assay demonstrated that Hsp90 binds to full-length LANA (**Figure 2B**). This experiment verified that N-terminal LANA associates with Hsp90.

Because the location of LANA is strictly limited to the nucleus, while Hsp90 is distributed in the cytoplasm but in virus infected cells has also been observed in the nucleus [55], we investigated whether both proteins co-localize. We used the KSHV positive endothelial tumor cell TIVE-L1 [23]. Cells were incubated with rabbit anti-LANA and mouse anti-Hsp90 antibodies and visualized using appropriate secondary antibodies (conjugated with Texas Red or FITC) (**Figure 2C**). LANA was located within nuclei of TIVE-L1 cells (Red) in the characteristic punctuate pattern. Part of Hsp90 was distributed within nuclei as previously described [56], and much of it in the cytoplasm (Green). A fraction of LANA and Hsp90 co-localized in the nucleus (Yellow). It is not clear at this point whether these co-localizing complexes represent functional episome tethering complexes or dead-end miss-folded accumulations.

### Hsp90 specific inhibitors disrupt the interaction between LANA and Hsp90

**Figure 3:**
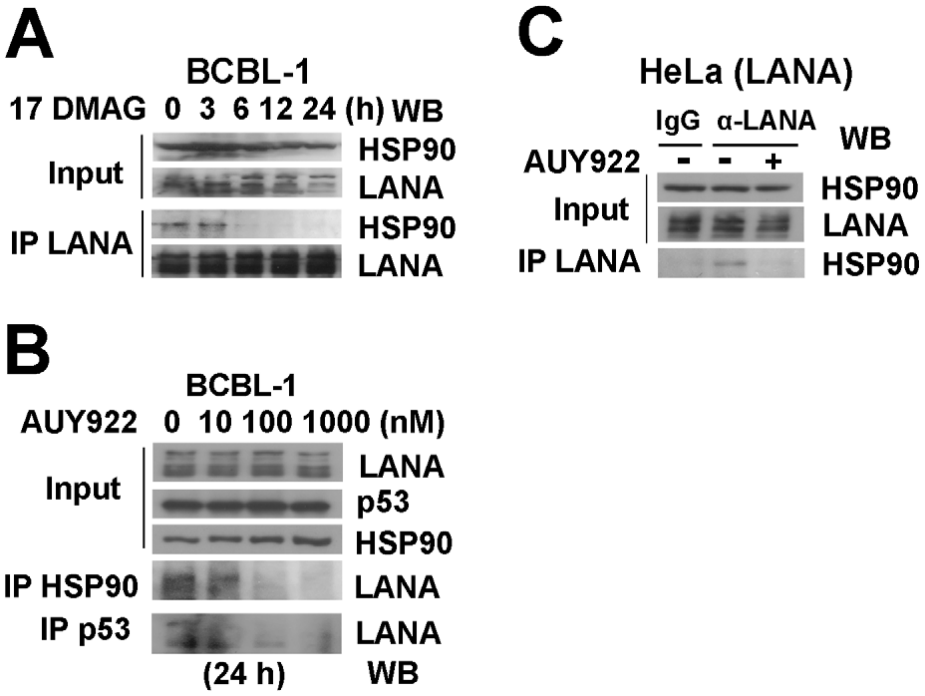
Hsp90 inhibitors disassociate LANA and Hsp90. (A) BCBL-1 cells were harvested after treatment with 0.5 µM 17-DMAG for 0, 3, 6, 12, 24 hours separately, cells lysates were immunoprecipitated with rat monoclonal anti-LANA antibody, followed by immunoblotting analysis with mouse anti-Hsp90 and anti-LANA antibodies. Input samples were from supernatants of lysed cells. (B) BCBL-1 cells were harvested after treatment with 0, 10, 100 and 1000 µM AUY922 separately for 24 hours, cell lyses were used for immunoprecipitation assay with anti-Hsp90 and anti-p53 antibodies, followed by immunoblotting analysis with anti-LANA. (C) Hela cells were transfected with LANA vector treated with no drug (DMSO) or 0.1 µM AUY922 for 24 hours. Immunoprecipitation was performed with rat anti-LANA antibody, followed by immunoblotting analysis with anti-LANA and anti-Hsp90 antibodies; IgG was used as control.

**Figure 4:**
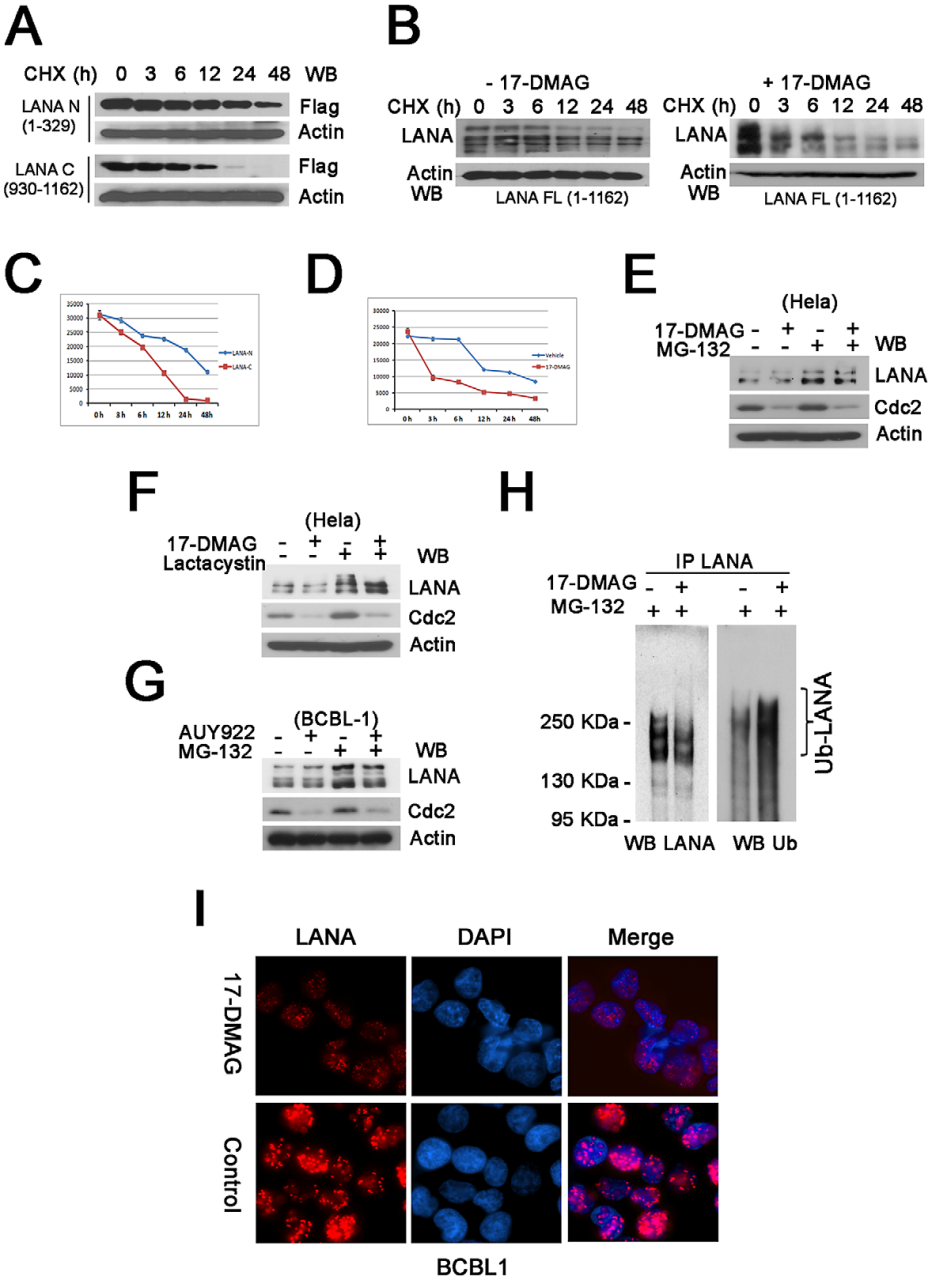
Hsp90 inhibitors induce proteasomal degradation of LANA. (**A**) Half-life analysis of LANA termini. N- or C- termini of Flag-tagged LANA vectors were transfected separately into HeLa cells for 16 hours, followed by cycloheximide (CHX) treatment for 0, 3, 6, 12, 24 and 48 hours. Whole cells lysates were immunoblotted with anti-Flag antibody. β-Actin was used for loading control. (**B**) LANA degradation induced by 17-DMAG. HeLa cells were transfected with full-length LANA overnight, followed by incubation with vehicle or 0.5 µM 17-DMAG in the presence of 50 µg/ml cycloheximide for 0, 3, 6, 12, 24 and 48 hours respectively, whole cells lysates were immunoblotted with anti-LANA. (**C–D**) Quantitative analysis of the above results. (**E–F**) LANA degradation inhibited by MG-132 or Lactacystin. After transfection with LANA vector, Hela cells were treated with no drug or 17-DMAG (1 µM) for 24-hours in the absence (−) or presence (+) of proteasome inhibitor MG-132 (10 µM) for the last 6 hours or Lactacystin (10 µM) for 24 hours, whole cells lyses were immunoblotted with anti-LANA antibody. (**G**) Proteasomal degradation of LANA in BCBL-1 cells. BCBL-1 were treated for 24 hours with no drug or AUY922 (0.1 µM) in the absence (−) or presence (+) of proteasome inhibitor MG-132 (10 µM) for the last 6 hours, whole cells lysates were immunoblotted with anti-LANA antibody. (**H**) Poly-ubiquitinated degradation of LANA. HeLa cells were transfected with LANA, followed by treatment with no drug or 1 µM 17-DMAG for 24 hours in the presence of DMSO or 10 µM MG-132 for the last 6 hours. Cells lysates were immunoprecitated with rat anti-LANA antibody, followed by immunoblotting with rabbit anti-ubiquitin and anti-LANA antibodies, the bracket shows the poly-ubiquitinated LANA (Ub-LANA). (**I**) Immunofluorescence analysis of LANA degradation. L1T2 cells were treated with no drug or 1 µM 17-DMAG for 48 hours, incubated with primary anti-LANA (rabbit) and after fixation and permeabilization, stained with anti-rabbit Texas-Red conjugated antibodies (red, LANA), nuclei were stained with DAPI.

To query the functional significance of the LANA-Hsp90 interaction, we used chemical inhibitors of Hsp90. The Hsp90 inhibitor, 17-dimethylamino-ethylamino-17-demethoxygeldanamycin (17-DMAG), disrupts Hsp90-client complexes, and reduces client protein levels, e.g. REV1, BCL6, or FANCA, through subsequent proteasomal degradation [56], [57], [58]. We hypothesized that 17-DMAG could similarly disrupt the interaction between LANA and Hsp90. To test this hypothesis, we treated BCBL-1 cells with 0.5 µM 17-DMAG at 0, 3, 6, 12, 24 hours, then immunoprecipitated LANA using a rat monoclonal antibody followed by immunoblotting analysis with anti-Hsp90 antibody. LANA disassociated from Hsp90 after incubation with 17-DMAG within 6 hours (**Figure 3A**). At 24 hours, we observed for the first time a reduction in LANA input levels, preferentially in the lower bands. This is expected because of the long half-life of LANA. More pronounced effects on overall LANA levels are only seen after 48 hours (Figure 4). The timing of cytotoxic inhibitor experiments is somewhat difficult as we are trying to measure a biochemical effect at the highest inhibition of Hsp90, but at a time where cells are not already dead.

To confirm the 17-DMAG results we used the new highly specific, ATP-competitive inhibitor of Hsp90 AUY922 [54], [59], [60], [61], [62], [63]. BCBL-1 cells were treated with AUY922 for 24 hours at increasing concentrations, followed by immune precipitation using anti-Hsp90 antibody and immunoblotting with anti-LANA antibody. AUY922 disrupted the LANA-Hsp90 complexes in BCBL-1 cells at 10–100 nM (**Figure 3B**). We and others had previously shown that LANA bound p53 [46], [48], [64]. As expected the LANA:p53 complexes were also diminished in the same concentration range.

To show independence of these interactions from other viral proteins and viral DNA we performed transient transfections. HeLa cells were transfected with a LANA expression vector for 24 hours after which AUY922 was added for 5 hours post-transfection. Again the Hsp90 inhibitor disassociated Hsp90 from LANA complexes (**Figure 3C**). In these experiments non-specific IgG was used as control. This demonstrates that functional inhibition of Hsp90 results in the disruption of the Hsp90-LANA complex.

### Hsp90 inhibitors induce proteasomal degradation of LANA

17-DMAG is known to accelerate degradation of Hsp90 client proteins [57], [58]. To test the hypothesis that 17-DMAG had a similar effect on the stability of LANA we monitored LANA protein levels after blocking de novo protein synthesis with cycloheximide (CHX). Since Hsp90 binds to the N-terminal of LANA but not the C-terminal (**Figure 2A**), we first determined the half-life of N- and C- terminal LANA proteins. Using transient transfection in Hela cells, we determined that the N-terminal domain of LANA was significantly more stable than the C-terminal domain of LANA, (**Figure 4A**), consistent with our conjecture that Hsp90 binding to the N-terminal domain contributed to overall stability. Next, we compared the half-life of transiently transfected full-length LANA after treatment with 17-DMAG to treatment with vehicle. 17-DMAG reduced the half-life of LANA by several hours compared to vehicle control (**Figure 4B**) while not affecting actin levels. These data were quantitated as shown in Figure 4, panel C and D. This establishes LANA as a client protein of Hsp90.

How was LANA degraded after Hsp90 inhibition? LANA protein accumulated after treatment with the proteasomal inhibitors Lactacystin and MG-132 in the presence of 17-DMAG (**Figure 4E–F**). As a control we used cdc2, which is an established client protein of Hsp90 [65]. MG-132 also increased in endogenous LANA levels in the BCBL-1 PEL cell line after treatment with AUY922 (**Figure 4G**). LANA levels were not affected by the autophagy inhibitor 3-Methyladenine (data not shown). These experiments are difficult, as they require titration of two drugs against two proteins, cdc2 and LANA, with different half-lives and differing dependencies on Hsp90. Nevertheless they suggest that LANA like other Hsp90 client proteins is degraded by the proteasome pathway.

To independently confirm these experiment we investigated LANA poly-ubiquitinylation in response to 17-DMAG, which represents one hallmark of entry into the proteasomal degradation pathway. Cell lysates of full length LANA plasmid-transfected HeLa cells treated with 17-DMAG or vehicle control in the presence MG-132 were used for immunoprecipitation with anti-LANA antibody. Immunoprecipitates were subjected to SDS-PAGE followed by immunoblotting with anti-LANA or anti-ubiquitin antibody. Of note LANA itself is a very large protein and runs at the top of even low-percentage SDS-PAGE gels. Some ubiquitinated LANA was present in cells after treatment with MG132 alone, but Hsp90 inhibition dramatically increased the poly-ubiquitination of LANA, as detected by a smear in the presence of 17-DMAG (**Figure 4H**). This demonstrates that Hsp90 targets miss-folded LANA for degradation through the ubiquitin-based proteasome pathway.

Inhibition of Hsp90 changed the characteristic nuclear punctuate pattern of LANA. When we added 17-DMAG in L1T2 cells for 48 hours at a concentration of 0.5 µM, LANA specific staining changed from a punctuate pattern into smaller dots irregularly distributed throughout the nucleus (**Figure 4I**). This result confirms our biochemical experiments and suggests the possibility that Hsp90 activity is required to maintain multimeric LANA complexes.

To determine whether Hsp90 inhibitors affect LANA transcription, we examined mRNA levels of LANA. BC-3, BCBL-1, BCP-1 and BC-1 cells were treated with 0.5 µM 17-DMAG for 0, 12 and 24 hours, and mRNA levels were measured by real-time qPCR. Relative expression was computed by comparison to the housekeeping gene GAPDH. The mRNA levels of LANA were unchanged upon Hsp90 inhibition (data not shown). We also examined the mRNA levels of RTA, an essential immediate early gene of KSHV. RTA levels also were unchanged. This demonstrated that LANA and Rta were not influenced by inhibition of Hsp90 at the transcriptional level, which implies that the reduction in LANA protein levels is not caused by transcriptional repression after drug treatment.

### The repeat sequence of the LANA central domain is dispensable for Hsp90 action

**Figure 5:**
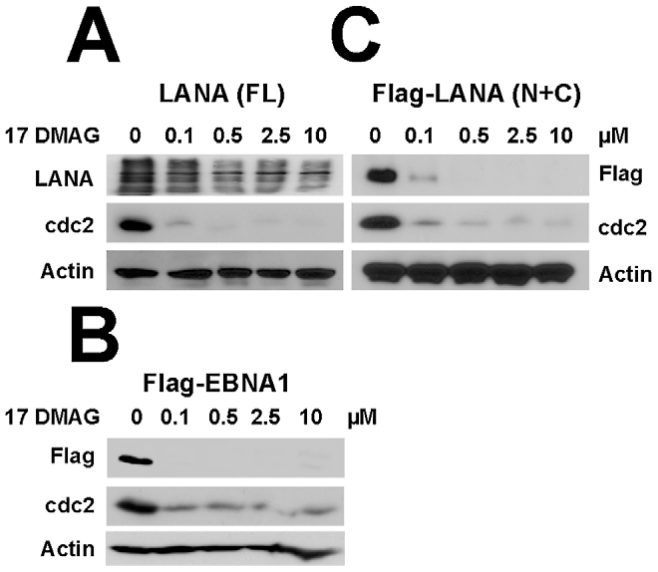
Central repeat domain of LANA resists degradation. (A–C) HeLa cells were transfected with plasmids including full-length LANA, LANA mutant (deleted repeat central domain) and EBNA1 respectively in six-well plates, followed by treatment with 17-DMAG at concentrations of 0, 0.1, 0.5, 2.5 and 10 µM for 48 hours. Whole cells lysates of each sample were immunoblotted with anti-LANA and anti-Flag antibodies, Cdc2 and β-Actin were used as controls.

Epstein-Barr Virus (EBV) encodes a functional, but not sequence homolog to LANA, the EBV nuclear antigen 1 (EBNA1). Both proteins have many characteristics in common: both are responsible for tethering the viral episome to host DNA in infected cells, and both proteins have unique central repeat domain that links the N-terminal to the C-terminal DNA binding domain. EBNA1 contains a Gly-Ala repeat, which mediates the Hsp90 enhancement of EBNA1 expression [66]. LANA has an acidic QED-rich repeat central repeat (CR) region that serves as the connector. Therefore we compared the effect of Hsp90 inhibition on LANA to EBNA1 in transiently transfected HeLa cells. LANA protein levels decreased gradually in a dose-dependent mode after treatment with 17-DMAG for 48 hours. Here, cdc2 was chosen as a cellular control, as it is a known substrate of Hsp90 [65] (**Figure 5A**). EBNA1 protein levels were also rapidly reduced even at very low concentrations of 17-DMAG (**Figure 5B**). Importantly, protein levels of a LANA mutant in which the acidic central repeat was deleted (LANA Mu (N+C)) were also diminished after treatment with 17-DMAG (**Figure 5C**). We used actin as a loading control and, cdc2 as control for Hsp90 inhibition. This demonstrates that the central region of LANA does not mediate Hsp90 interaction. It is consistent with our mapping data, which showed that Hsp90 bound the N-terminal domain of LANA. It suggests that the molecular mechanism of Hsp90-mediated stabilization of LANA differs from that of Hsp90-mediated stabilization of EBNA1.

### Hsp90 inhibitors have therapeutic potential against PEL

**Figure 6:**
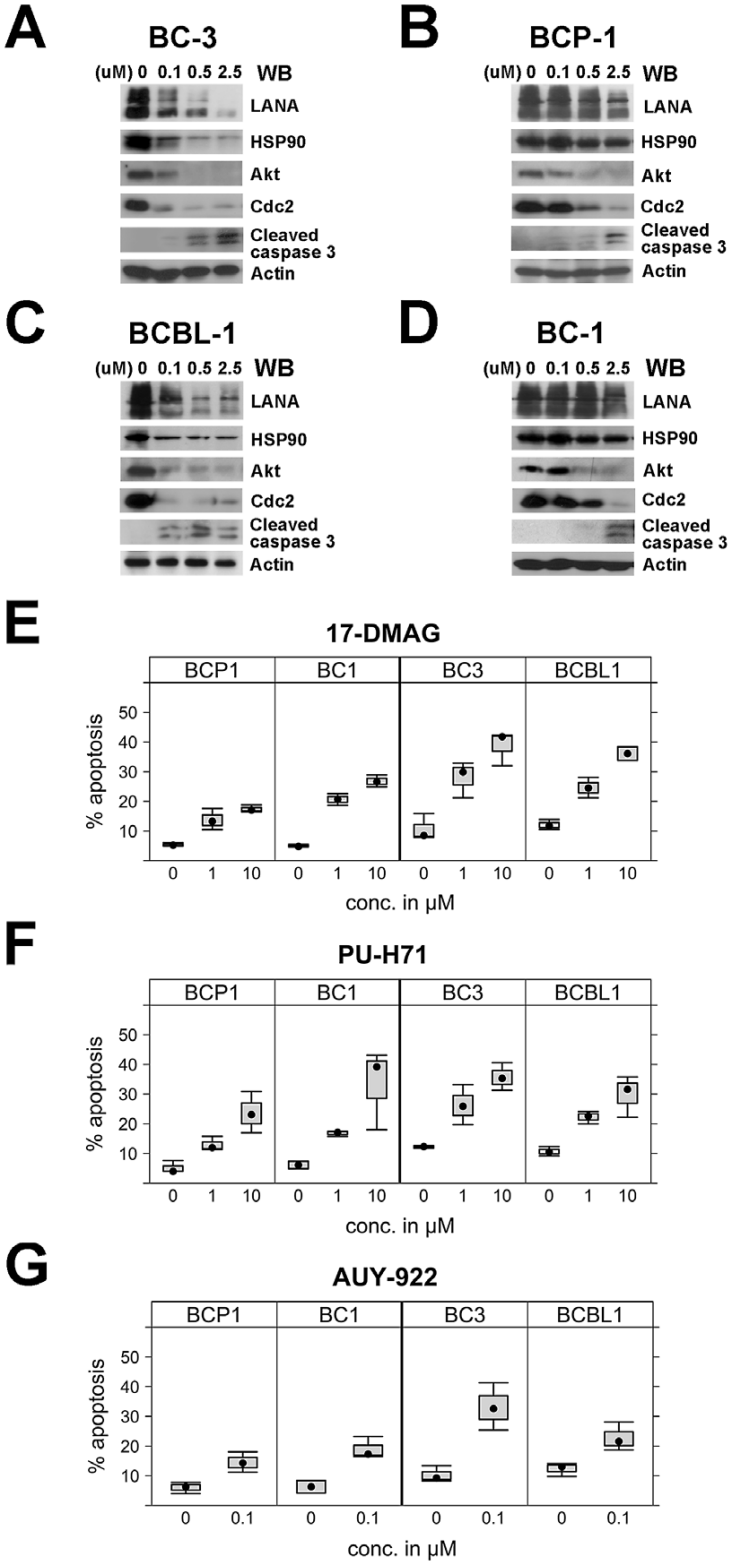
Effects of Hsp90 inhibitors on LANA in PEL cells. (A–D) Hsp90 inhibitors repress LANA expression in PEL cells. PEL cells including BC-3, BCP-1, BCBL-1, and BC-1 cells were treated with 17-DMAG at concentrations of 0, 0.1, 0.5, 2.5 and 10 µM and after 48 hours, whole cells lysates were immunoblotted with anti-LANA, anti-Hsp90, anti-Akt, and anti-cleaved caspase-3 antibodies, Cdc2 and β-Actin were used as controls. (**E–G**) Apoptosis. PEL cells including BC-3, BCP-1, BCBL-1, and BC-1 cells were treated respectively with Hsp90 inhibitors 17-DMAG and PU-H71 at concentrations of 0, 1, and 10 µM, and 10 µM, or 0, 0.1 µM AUY922 for 24 hours, apoptosis percentage was analyzed after PEL cells were harvested and stained.

Having demonstrated that Hsp90 was an important molecular chaperone of LANA, we explored the potential of Hsp90 inhibitors as anti-PEL tumor therapeutics. We used cleaved caspase-3 as a marker for cell death. We treated PEL cells with the Hsp90 inhibitor 17-DMAG at different concentrations (0, 0.1, 0.5 and 2.5 µM) for 48 hours. BC-3 and BCBL-1 cells were more sensitive to 17-DMAG compared to BCP-1 and BC-1. The appearance of cleaved caspase-3 as a marker of apotosis was at lower concentrations 500 nM and 100 nM in BC-3 and BCBL-1, respectively (**Figure 6** **A and C**). LANA expression, too, was readily diminished at sub-micromolar concentrations of the inhibitor. Apoptosis in PEL involves p53 and this phenotype correlated with p53 status [67]. BC3 and BCBL-1 have wild-type functional p53 and were more sensitive to 17-DMAG, BCP-1 and BC-1 have mutant p53 and were less sensitive to 17-DMAG. Of course, p53 status is not the only difference among these [68]. They required 2.5 µM 17-DMAG to induce caspase-3 cleavage.

As an additional cellular Hsp90 control we investigated Akt, which is a known client protein of Hsp90. Akt and Akt/mTOR signaling is required for PEL growth [51], [69]. Akt was decreased in all PEL cells in a dose-dependent manner after 17-DMAG treatments as was cdc-2. Again, in BC-3 and BCBL-1 cdc-2 expression was abrogated at 100 nM inhibitor, whereas 500–2500 nM were needed to show a similar downregulation of cdc-2 in BCP-1 and BC-1 cells (**Figure 6 A–D**). In sum, multiple Hsp90 client proteins are degraded upon exposure of PEL to 17-DMAG, many of which (LANA, K1 [50], Akt) with known oncogenic roles in PEL tumorigenesis.

To extend our observations with regard to the therapeutic potential of Hsp90 inhibitors for PEL, we treated multiple PEL cell lines with three different Hsp90 inhibitors at different concentrations for 24 hours as indicated and measured apoptosis by flow cytometry for annexin V (**Figure 6E–G**). We used 17-DMAG, AUY922 and a third, novel ATP-competitive Hsp90 inhibitor PU-H71 [70]. All induced apoptosis in a dose-dependent fashion (p≤5*10^−16^, by ANOVA). The p53 wild type BC-3 was the most sensitive and the p53 mutant BCP-1 the least sensitive cell line independent of drug and concentration (p≤0.015 for BCP-1, p≤0.003 for BC3, based on ANOVA. The difference between BCP-1 and BC3 was p≤0.000001 based on Tukey-HSD test). BC-3 cells showed 38.7% apoptosis while BCP-1 cells showed only 18% apoptosis when treated with 10 µM 17-DMAG. All PEL lines seemed more sensitive to AUY922 than to the other two drugs, though this did not reach a level of statistical significance at a 95% family-wise confidence level (**Figure 6F**). As with all chemical inhibitor studies we cannot exclude that differential sensitivity is a function of different drug entry and efflux from cell. In sum, established and novel Hsp90 inhibitors inhibit cell growth and apoptosis in PEL cells.

### Sh-RNA mediated knockout of Hsp90 leads to PEL apoptosis

**Figure 7:**
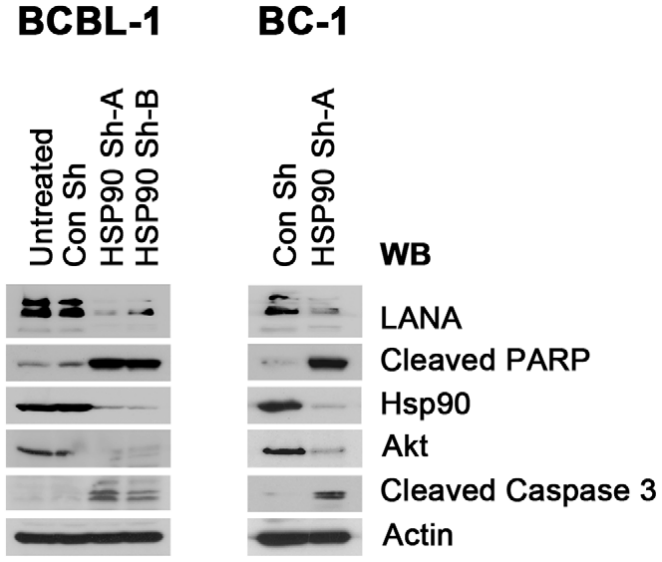
Effects of Hsp90 on LANA after lentiviral knockout. BCBL-1 and BC-1 cells were infected with recombinant lentiviruses targeting Hsp90 in six-well plates; empty lentivirus vector and untreated cells were used as controls. Samples were harvested after 4 days and immunoblotted with anti-LANA, anti-Hsp90, and anti-Akt antibodies, respectively. Apoptosis was evaluated by immunoblotting assay with anti-cleaved PARP and Caspase-3 antibodies separately. β-Actin was used as loading control.

To guard against the possibility of off target effects of chemical Hsp90 inhibitors, we used recombinant lentiviruses. Two vectors, Sh-A and Sh-B, which target Hsp90 were transduced into BCBL-1; empty lentivirus or untreated cells were used as controls. Hsp90 protein levels were dramatically reduced compared to untreated cells upon specific shRNA transduction with either sh-A or sh-B, but not irrelevant control (**Figure 7**). Upon depletion of Hsp90, the protein levels of LANA and the host control client protein Akt were decreased compared to controls. Lentivirus Sh-A was slightly more efficient than Sh-B and was also used in BC-1 cells with the same result: upon reduction of Hsp90, the level of LANA decreased as well. At the same time, expression levels of both cleaved PARP and Caspase-3 were increased indicative of apoptosis. This demonstrates that Hsp90 is essential for the survival of PEL and that direct inhibition of Hsp90 rather than off target effect of the drugs mediate the therapeutic efficacy of Hsp90 inhibitors against PEL.

### Hsp90 inhibitors inhibit KS tumor growth and reduce ephrin-B2 and EphA2 levels

**Figure 8:**
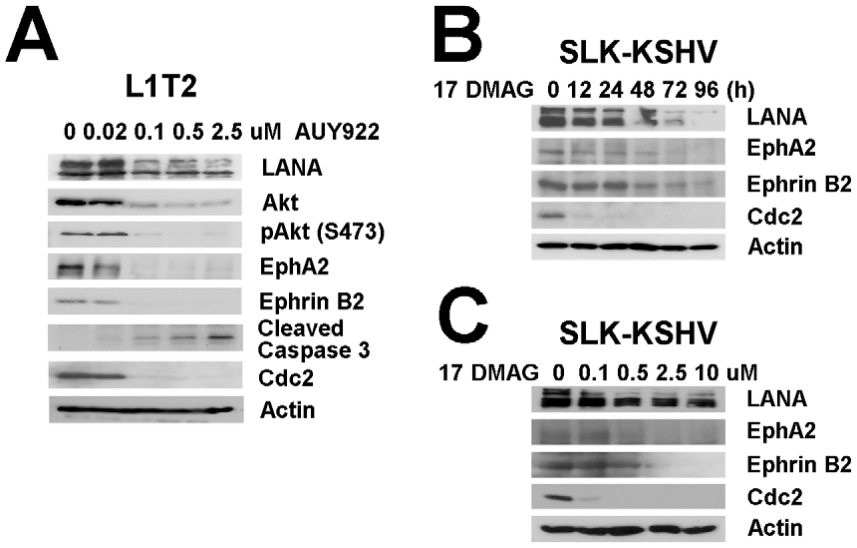
Effects of Hsp90 inhibitors on LANA in KSHV-infected endothelial cells. (A) L1T2 cells were treated with AUY922 at concentrations of 0, 0.02, 0.1, 0.5, and 2.5 µM for 48 hours, whole cell lysates were immunoblotted with anti-LANA, anti-Hsp90 antibodies, anti-EphA2, anti-Ephrin-B2, and anti-Akt (total), and anti-pAkt (S473) antibodies separately. Apoptosis was evaluated with anti-cleaved PARP and anti-cleaved Caspase-3 antibodies, Cdc2 and β-Actin were used as controls. (B–C) SLK-KSHV were treated with 0.5 µM 17-DMAG for 0, 12, 24, 48, 72 and 96 hours, or at concentrations of 0, 0.1, 0.5, 2.5 and 10 µM for 48 hours separately, whole cell lysates were immunoblotted with anti-LANA antibody, anti-EphA2, anti-EphrinB2, Cdc2 and β-Actin were used as controls.

In addition to PEL, which is a B cell lymphoma, KSHV is also associated with the development of KS, an endothelial lineage tumor. To explore the potential of Hsp90 inhibitors as novel anti-KS therapeutics we used KS culture and animal models. The L1T2 cell line was established from KSHV positive L1-TIVE cells [23]. It is more aggressive than the parent line and readily induces tumors in SCID mice (Roy and Dittmer, submitted). L1T2 cells were treated with increasing doses of AUY922 for 48 hours (**Figure 8A**). Immunoblotting confirmed that LANA protein levels were decreased in a dose-dependent manner. Cdc2 protein levels were used as control for Hsp90 inhibition and also decreased in a dose-dependent manner. Actin protein levels were used as control for loading and remained constant independent of the dose of AUY922. At the same concentration that cdc2 levels decreased, Akt, and phosphorylated Akt protein levels were decreased. This confirmed the specificity of the inhibitor for Hsp90. Cleaved Caspase-3 was increased. Similar results were observed in another KS cell model after treatment with a different Hsp90 inhibitor. SLK-KSHV [47] were treated with 17-DMAG with different dosages (0, 0.1, 0.5, 2.5 and 10 µM) and times (0, 12, 24, 48, 72 and 96 h) and LANA protein levels were reduced in a dose- and time-dependent manner (**Figure 8** **B–C**). Note that in this model cell growth is not dependent on LANA, which supports the notion of LANA as a direct target of Hsp90.

KS tumorigenesis is more complicated than PEL tumorigenesis in that KSHV re-infection seems to contribute to the transformed phenotype [47]. Recently, the EphA2 receptor tyrosine kinase was implicated as a co-receptor for KSHV [34], [35]. Hsp90 is an essential regulator of EphA2 stability [71]. Therefore, we tested the hypothesis that EphA2 is also a client protein of Hsp90 in KS. EphA2 expression was reduced in the two KS cell lines (L1T2, SLK-KSHV) after treatment with two different Hsp90 inhibitors (**Figure 8**). The reduction in EphA2 was both dose and time dependent, confirming that in KS, as in other cancers, EphA2 is a client of Hsp90.

**Figure 9:**
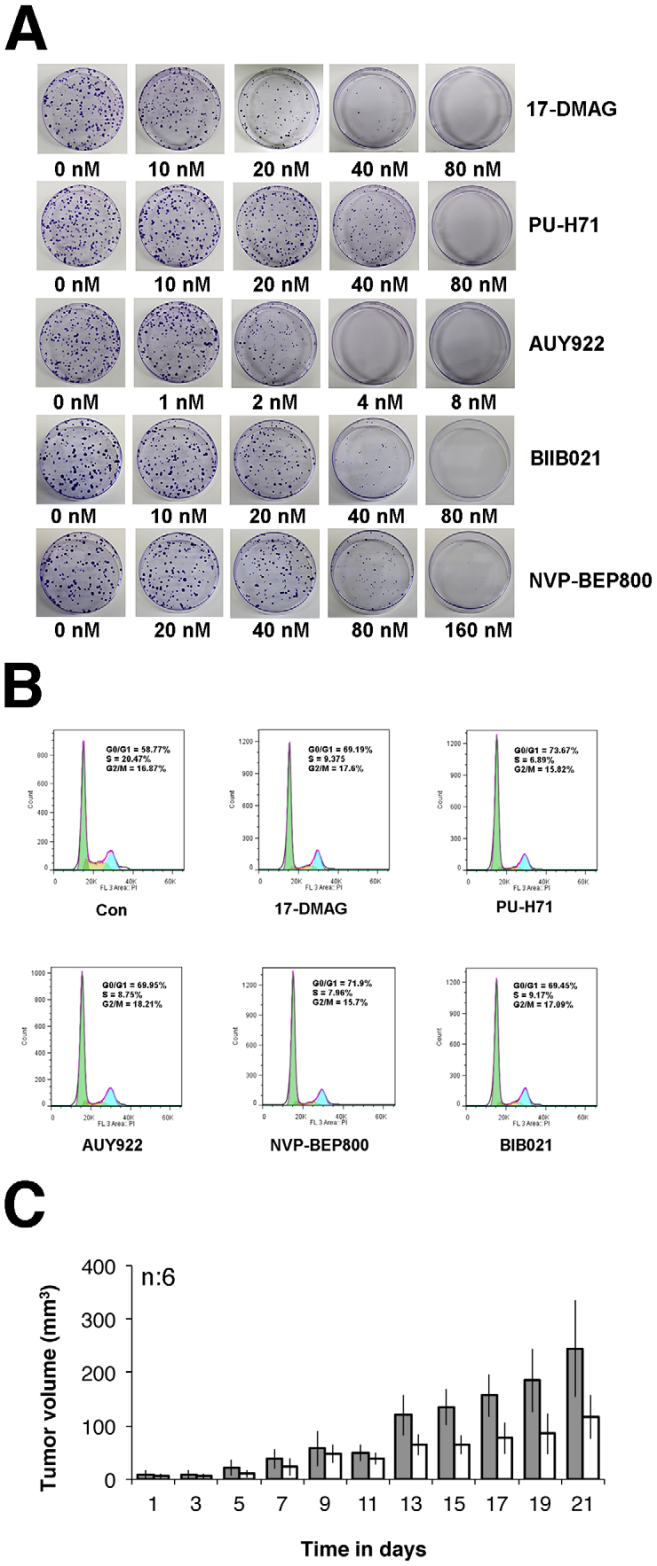
Hsp90 inhibitors repress KS tumor growth. (A) Colony formation assay of L1T2 cells after drug treatment. 400 L1T2 cells were seeded in 10 cm dishes, followed by addition of two-fold serially-diluted Hsp90 inhibitors; 17-DMAG, PU-H71, AUY922, BIIB021, and NVP-BEP800, respectively for two weeks. Colonies were counted after incubation with Magic Blue Staining, each experiment was repeated three times. (B) Hsp90 inhibitors induce G0/G1 arrest of L1T2 cells. L1T2 cells were treated with 0.5 µM of 17-DMAG, PU-H71, NVP-BEP800, BIB021, or 0.05 µM AUY922 for 24 hours, DMSO treatment was used as control. After cells were fixed and stained with propidium iodide, cell-cycle analysis was performed using flow cytometry. The percentages of cells at different stages in the cell cycle (G0/G1, S, G2/M) are shown. (C) Growth curves of tumor volume. 10^5^ L1T2 cells were injected sub-cutaneously into C.B.-17 SCID mice with mixed Matrigel (1∶1) for three days, followed by AUY922 intra-peritoneal injection at doses of 50 mg/kg NVP-AUY922 for total three weeks (three times per week), tumor volumes of SCID mice were measured and analyzed. Two groups were analyzed and each group had six mice, mock-treated mice were used as control.

KS also expresses ephrin-B2, but not its receptor EphB4. Ephrin-B2 is critical for the survival of KS tumor cells, while EphB4 is downregulated upon KSHV infection [19], [20], [72]. Therefore, we tested the hypothesis that ephrin-B2 is also affected by Hsp90 inhibition in KS. EphrinB2 protein levels were decreased in the different KS cell lines after treatment with Hsp90 inhibitors, in a dose- and time-dependent fashion (**Figure 8**). This is the first study implicating ephrin-B2 as a potential client of Hsp90. Similar to PEL before, we also found that total Akt protein levels and phosphorylated Akt (S473) were decreased in L1T2 cells upon exposure to AUY922. This correlated with a time dependent increase in the levels of cleaved PARP and caspase-3, which are markers of apoptosis (Figure 9A). This demonstrates that Hsp90 inhibition decreases essential viral (LANA) and host (EphA2, ephrin-B2, Akt) client protein levels in KS resulting in cell death.

### Hsp90 inhibitors repress proliferation of KS

To expand our observations we measured the effect of Hsp90 inhibitors on KS cell growth. First, we used the xCELLigence system to measure proliferation in real-time, and we added two additional Hsp90 inhibitors, BIIB021 and NVP-BEP800. SLK-KSHV, L1T2, SLK and KS-IMM were treated separately with 17-DMAG, PU-H71, AUY922, BIIB021 and NVP-BEP800. IC50 values were determined based on real-time growth curves using the XCelligence system (**Table 3**). All Hsp90 inhibitors had nanomolar IC50s. AUY922 was the most efficacious among these five drugs. It had single nanomolar or even sub-nanomolar IC50 against all cell lines, which was an order of magnitude lower than the IC50 for the other Hsp90 inhibitors. NVP-BEP800 was least effective, possibly due to a weak solubility [73].

**Table 3:** IC50 based on Xcelligence measurements of cell proliferation.

Drug/Cell	17-DMAG	PU-H71	AUY 922	BIIB021	NVP-BEP800
L1T2	19±3	22±8	2±0.2	29±4	93±8
KS-KMM	15±8	30±9	2±1	20±9	138±5
SLK	55±2	81±10	4±2	54±5	247±29
SLK- KSHV	11±5	±0.1	0.5±0.2	29±3	57±12
Ratio SLK-KSHV/SLK	3.3…9.5×	4.2…5.3×	2.8…20×	1.5…2.3×	3.2…6.2×

All concentrations are in nM and the result of a titration series with 7 drug concentrations each in n = 2 individual titration experiments. Also shown is the range of ratios for IC50s for SLK compared to isogenic SLK-KSHV cells. This can be interpreted as a selectivity index of KSHV positive compared to KSHV negative cells.

The results also indicated that every Hsp90 inhibitor was more effective in the KSHV-positive SLK cells compared to isogenic KSHV-negative SLK cells. This is quantified in table 3, which shows the range of ratios comparing the IC50 of SLK cells to SLK cells carrying KSHV. This demonstrates that KS/endothelial lineage tumors are exquisitely sensitive to Hsp90 inhibition and that part of this phenotype can be attributed to the presence of KSHV latent proteins.

To independently verify the potency of the Hsp90 inhibitors, we performed clonogenic colony formation assays. All drugs inhibited cell growth with nanomolar IC50s. AUY922 again was the most efficacious among the five drugs in these assays, with an IC50 of 2 nM (**Figure 9A**).

Third, we performed cell-cycle analysis. L1T2 cells were treated with 500 nM of 17-DMAG, PU-H71, BIIB021, NVP-BEP800, or 50 nM AUY922 for 24 hours and subjected to cell cycle profiling using propidium iodide staining. DMSO treatment was used as a control. The cells stopped cycling with a reduction in S phase, which was 20.47% for control and <9.5% for each of the five drug treated samples. At the same time the fraction of G0/G1 cells increased from 58.77% for control to 69.19%–73.67% in each of the five drug treated cells. AUY922 was as effective as the other four inhibitors even though it was used at 10 fold lower concentration (**Figure 9B**). In sum, Hsp90 inhibitors repress KS tumor cell proliferation at nanomolar concentrations.

To further investigate the anti-tumor activity of AUY922, we subcutaneously injected SCID mice with KSHV-infected L1T2 cells (10^5^/mouse) as previously published [53]. Upon the development of palpable tumors the mice were randomized to two groups and with AUY922 (50 mg/kg) for three weeks (three times per week) or vehicle. All the animals were sacrificed after 21 days as per IACUC stipulation. AUY922 significantly retarded tumor growth compared to the mock-treated mice (p≤0.05 by repeated measurements analysis of variance (ANOVA)) (**Figure 9C**).

**Figure 10:**
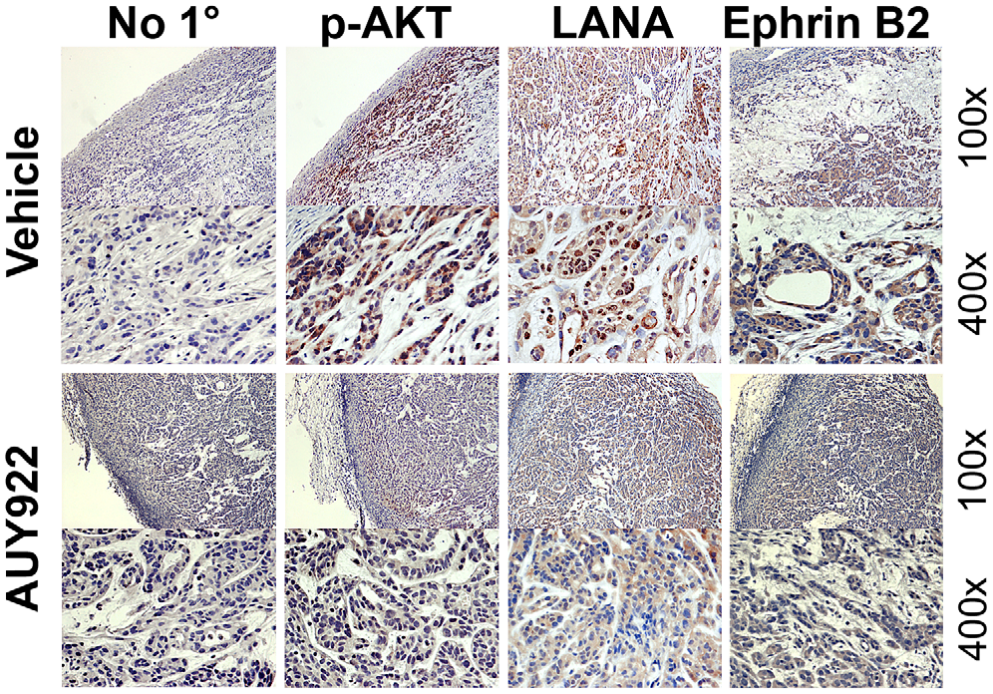
Immunohistochemistry analysis of mouse Xenograft tumors. Solid tumors were excised from mock or drug treated mice, followed by immunohistochemistry staining using antibodies specific for p-Akt, LANA, and Ephrin B2. No specific primary antibody was used for control. Images were taken at 100× and 400× magnification.

To demonstrate molecular activity of AUY922 in vivo, we measured Hsp90 client protein levels in the tumor grafts by immune histochemistry (**Figure 10**). No staining was observed without primary antibody. (i) As expected [74], [75] phosphorylated Akt was detectable in all viable tumor cells (excluding those in liquefied or necrotic areas). The phosphorylation level of Akt was substantially reduced after AUY922 treatment. (ii) LANA was detected in the nuclei of KS xenograft mouse tumors, and LANA levels were reduced after treatment. (iii) ephrin B2 expression was expressed at substantial levels in all KS cell lines and our immunohistochemical results detected ephrin B2, in vascular structures and tumor cells in KS xenograft tumors. Ephrin B2 levels were significantly decreased after AUY922 treatment. These experiments support the notion that LANA, AKT and ephrinB2 are bona fide targets of Hsp90 in KS tumors in vivo and provide proof-of-principle for the use of Hsp90 inhibitors as potential anti-KS therapeutics.

## Discussion

This study shows that KSHV LANA is a novel client protein of Hsp90. Hsp90 associates with the N-terminus of LANA. ATP-competitive Hsp90 inhibitors disrupt this interaction and reduce the half-life of LANA by accelerating ubiquitin-mediated, proteasomal degradation of LANA. LANA plays an essential role in KSHV genome persistence and KS tumorigenesis [38], [76]. Chemical inhibition of Hsp90 or Hsp90 depletion using shRNAs led to rapid apoptosis of KS tumor cells and inhibited KS xenograft growth in mice. In addition to LANA, we validated cdc2, Akt, EphA2 and ephrin-B2 as targets of Hsp90 in KS. Earlier studies identified additional Hsp90 clients in PEL [14], [15], [77]. This establishes Hsp90 as a novel target for anti-viral and anti-tumor strategies in KS and PEL.

The dependence on Hsp90 is shared between KSHV LANA and EBV EBNA1 [66]. Since LANA and EBNA-1 do not share sequence similarity, yet they are structural and functional homologs, the mechanism of Hsp90 interactions differs for both proteins. In case of EBNA1, the central Gly-Ala repeat domain is required for Hsp90 inhibition [66]; in the case of LANA the N-terminal domain mediates the Hsp90 interaction, though the central repeat region may contribute to overall stability as well. EBNA1 is degraded through autophagy after Hsp90 inhibition; LANA was degraded through the ubiquitin/proteosome pathway. There is also the question of cellular localization. Sun et al. [66] did not find a direct EBNA1:Hsp90 interaction and consequently did not query where the EBNA1:Hsp90 interaction took place. They focused their efforts on EBNA1 translation and elegantly showed that translation of the Gly-Ala repeat required Hsp90 in an in vitro translation reaction. Our studies show that LANA affected overall stability of LANA, but also evidence for a nuclear interaction. Hsp90 can be present in both the cytoplasm and the nucleus [78], [79], [80], possibly fulfilling different roles in either compartment. Most recently nuclear BRCA1 and DNA-PKA were validated as novel client proteins of Hsp90 [81], [82], which implicates Hsp90 in the DNA damage/repair response. Regardless of mechanism, the LANA:Hsp90 interaction can be exploited to kill KSHV-associated tumors.

Hsp90 inhibitors represent promising drugs for cancer therapy and many have advanced into phase I clinical trials. We previously implicated the Hsp90 inhibitor 17-DMAG as a chaperone for the KSHV K1 protein and showed that it had activity against PEL cells [50]. 17-DMAG and the related compounds 17-AAG/Tanespimycin and geldanamycin had varying efficacy in early clinical trials, due to toxicity, choice of target cancer type, and perhaps because these compounds are substrates for the P-glycoprotein efflux pump and have sub-optimal pharmacokinetics in humans (reviewed in [8]). In addition Hsp90 fulfills crucial functions in normal cells, in the EBV life cycle [66], and in fact the lytic replication of other viruses (reviewed in [83], [84], [85]). Therefore it has been a concern that very potent Hsp90 inhibitors would affect basic cell functions non-specifically and that therefore their selectivity index would be low. For instance, Hsp90 has been implicated in cardiac potassium channel maturation; yet cardiac toxicity has not emerged as dose limiting in phase I trials. 17-DMAG and other benzoquinone derivative cause liver toxicity. That phenotype was not related to Hsp90 inhibition and prompted the screen for second-generation Hsp90 inhibitors, which we explored here. Another potential application is, at least hypothetically, the treatment of neurodegenerative diseases, which result in the accumulation of miss-folded proteins. The requirement for Hsp90 in cancer cells, virally infected cells or cells that accumulate misfolded proteins seems to be so profound that it translates into selectivity in clinical settings for second generation Hsp90 inhibitors; alternatively it has been suggested that the hsp90 multi-protein complex differs between tumor cells and normal cells and that this would result in increased drug access to the Hsp90 ATP binding sites. To date over 20 different Hsp90 inhibitors have passed pre-clinical toxicity studies and advanced into phase I clinical trials [85].

Our studies went beyond the first generation 17-DMAG/17-AAG/geldanamycin structural class of hsp90 inhibitors and evaluated four new, fully synthetic, chemically distinct ATP-competitive inhibitors: PU-H71, AUY922, BIIB021, BEP800. All inhibited KS and PEL tumor growth at low nanomolar concentrations and all decreased the levels of other, known Hsp90 client proteins such as cdc2 and Akt [5]. Whereas all PEL were susceptible to Hsp90 inhibitors, we did observe cell line variation. This is expected since these PEL cell lines have accumulated both common and cell line specific genomic alterations [68]. We and others observed similar alterations to other targeted drugs previously [51], [64], [67], [69], some of the variation could be explained by p53 status, other drug-specific variation has yet to be identified. This is a common effect seen in almost all studies that use panels of cell lines rather than a single cell line as read-out. AUY922 had the lowest IC50 (2 nM) against a battery of KS cell lines. It is a product of structure-guided optimization of 4, 5-diarylisoxazole compounds, which block the ATP-binding pocket of Hsp90 [54], [86]. AUY922 inhibited a tumor growth in a xenograft KSHV tumor model with similar efficacy as reported previously for other anti-KS compounds [54]. Recent studies have demonstrated that, as a small-molecule inhibitor, AUY922 exhibits promising therapeutic potential in a variety of cancers as such as lung cancer, glioblastoma, myeloma, etc. [59], [60], [61], [63]. KS and PEL can now be added to the list and should be included in early-phase clinical explorations of this compound.

It is likely that the pronounced anti-tumor effect of Hsp90 inhibitors is due to the downregulation of multiple targets: LANA, which is essential for viral maintenance [17], cdc2, Akt, which transduces paracrine and autocrine growth signals in PEL, KS and other cancers [51], NFkB activators [15], ephrin-B2, and EphA2, which support KSHV re-infection of endothelial cells and thus tumor maintenance and even targets of surface bound Hsp90 [16].

Ephrins and Ephrin receptors are key molecules in endothelial cell proliferation, tumorigenesis, and essential co-factors for KSHV infection [34], [35]. Ephrin receptor tyrosine kinases and their ephrin ligands transduce signals in cell-cell contact-dependent fashion. Their expression in endothelial cells promotes angiogenesis [18], [87], [88]. We found two different molecules in this network to be client proteins of Hsp90 in KS: EphA2 and ephrin-B2 The EphA2 receptor kinase was previously identified as an Hsp90 client [29], [30]. Our studies showed that EphA2 was expressed abundantly in L1T2, SLK-KSHV, and KS-IMM cells and that Hsp90 inhibitors reduced EphA2 expression. Ephrin-B2 also plays multiple roles in vessel maturation, and is expressed at substantial levels in KS [19], as well as in the KS tumor models we examined in this study. Infection of endothelial cells with KSHV induces expression of Ephrin-B2, and Ephrin B2 is required for KS survival [19]. Blockage of Ephrin-B2 signaling with the extracellular domain of EphB4 fused with human serum albumin (sEphB4-HSA), suppressed a wide variety of tumors including KS [19], [20], [31], [89], [90]. We found that Hsp90 inhibitors significantly decreased the expression of Ephrin-B2 in multiple KS tumor models (L1T2, SLK-KSHV), which suggests that downregulation of ephrin interactions through Hsp90 inhibitors contributes to their effectiveness in the endothelial lineage tumor KS.

## References

[1] Zhao R, Davey M, Hsu YC, Kaplanek P, Tong A, et al (2005) Navigating the chaperone network: an integrative map of physical and genetic interactions mediated by the hsp90 chaperone. Cell 120: 715–727. 15766533 10.1016/j.cell.2004.12.024

[2] Taipale M, Jarosz DF, Lindquist S (2010) HSP90 at the hub of protein homeostasis: emerging mechanistic insights. Nat Rev Mol Cell Biol 11: 515–528. 20531426 10.1038/nrm2918

[3] Calderwood SK, Khaleque MA, Sawyer DB, Ciocca DR (2006) Heat shock proteins in cancer: chaperones of tumorigenesis. Trends Biochem Sci 31: 164–172. 16483782 10.1016/j.tibs.2006.01.006

[4] Whitesell L, Lindquist SL (2005) HSP90 and the chaperoning of cancer. Nat Rev Cancer 5: 761–772. 16175177 10.1038/nrc1716

[5] Moulick K, Ahn JH, Zong H, Rodina A, Cerchietti L, et al (2011) Affinity-based proteomics reveal cancer-specific networks coordinated by Hsp90. Nat Chem Biol 7: 818–826. 21946277 10.1038/nchembio.670 PMC3265389

[6] McClellan AJ, Xia Y, Deutschbauer AM, Davis RW, Gerstein M, et al (2007) Diverse cellular functions of the Hsp90 molecular chaperone uncovered using systems approaches. Cell 131: 121–135. 17923092 10.1016/j.cell.2007.07.036

[7] Wandinger SK, Richter K, Buchner J (2008) The Hsp90 chaperone machinery. J Biol Chem 283: 18473–18477. 18442971 10.1074/jbc.R800007200

[8] Trepel J, Mollapour M, Giaccone G, Neckers L (2010) Targeting the dynamic HSP90 complex in cancer. Nat Rev Cancer 10: 537–549. 20651736 10.1038/nrc2887 PMC6778733

[9] Jego G, Hazoume A, Seigneuric R, Garrido C (2010) Targeting heat shock proteins in cancer. Cancer Lett E-pub ahead of print. 10.1016/j.canlet.2010.10.014 21078542

[10] Wang RE (2011) Targeting Heat Shock Proteins 70/90 and Proteasome for Cancer Therap. Curr Med Chem 18: 4250–64. 21838681 10.2174/092986711797189574

[11] Prodromou C, Roe SM, O'Brien R, Ladbury JE, Piper PW, et al (1997) Identification and structural characterization of the ATP/ADP-binding site in the Hsp90 molecular chaperone. Cell 90: 65–75. 9230303 10.1016/s0092-8674(00)80314-1

[12] Banerji U (2009) Heat shock protein 90 as a drug target: some like it hot. Clin Cancer Res 15: 9–14. 19118027 10.1158/1078-0432.CCR-08-0132

[13] Chen G, Cao P, Goeddel DV (2002) TNF-induced recruitment and activation of the IKK complex require Cdc37 and Hsp90. Molecular cell 9: 401–410. 11864612 10.1016/s1097-2765(02)00450-1

[14] Higashi C, Saji C, Yamada K, Kagawa H, Ohga R, et al (2012) The effects of heat shock protein 90 inhibitors on apoptosis and viral replication in primary effusion lymphoma cells. Biological & pharmaceutical bulletin 35: 725–730. 22687408 10.1248/bpb.35.725

[15] Field N, Low W, Daniels M, Howell S, Daviet L, et al (2003) KSHV vFLIP binds to IKK-gamma to activate IKK. Journal of cell science 116: 3721–3728. 12890756 10.1242/jcs.00691

[16] Qin Z, DeFee M, Isaacs JS, Parsons C (2010) Extracellular Hsp90 serves as a co-factor for MAPK activation and latent viral gene expression during de novo infection by KSHV. Virology 403: 92–102. 20451233 10.1016/j.virol.2010.03.052 PMC3202419

[17] Godfrey A, Anderson J, Papanastasiou A, Takeuchi Y, Boshoff C (2005) Inhibiting primary effusion lymphoma by lentiviral vectors encoding short hairpin RNA. Blood 105: 2510–2518. 15572586 10.1182/blood-2004-08-3052

[18] Kuijper S, Turner CJ, Adams RH (2007) Regulation of angiogenesis by Eph-ephrin interactions. Trends Cardiovasc Med 17: 145–151. 17574121 10.1016/j.tcm.2007.03.003

[19] Masood R, Xia G, Smith DL, Scalia P, Still JG, et al (2005) Ephrin B2 expression in Kaposi sarcoma is induced by human herpesvirus type 8: phenotype switch from venous to arterial endothelium. Blood 105: 1310–1318. 15471957 10.1182/blood-2004-03-0933

[20] Scehnet JS, Ley EJ, Krasnoperov V, Liu R, Manchanda PK, et al (2009) The role of Ephs, Ephrins, and growth factors in Kaposi sarcoma and implications of EphrinB2 blockade. Blood 113: 254–263. 18836096 10.1182/blood-2008-02-140020 PMC2614637

[21] Wang L, Damania B (2008) Kaposi's sarcoma-associated herpesvirus confers a survival advantage to endothelial cells. Cancer Research 68: 4640–4648. 18559509 10.1158/0008-5472.CAN-07-5988 PMC2612117

[22] DiMaio TA, Gutierrez KD, Lagunoff M (2011) Latent KSHV infection of endothelial cells induces integrin beta3 to activate angiogenic phenotypes. PLoS pathogens 7: e1002424. 22174684 10.1371/journal.ppat.1002424 PMC3234222

[23] An FQ, Folarin HM, Compitello N, Roth J, Gerson SL, et al (2006) Long-term-infected telomerase-immortalized endothelial cells: a model for Kaposi's sarcoma-associated herpesvirus latency in vitro and in vivo. Journal of Virology 80: 4833–4846. 16641275 10.1128/JVI.80.10.4833-4846.2006 PMC1472065

[24] Hong YK, Foreman K, Shin JW, Hirakawa S, Curry CL, et al (2004) Lymphatic reprogramming of blood vascular endothelium by Kaposi sarcoma-associated herpesvirus. Nature genetics 36: 683–685. 15220917 10.1038/ng1383

[25] Flore O, Rafii S, Ely S, O'Leary JJ, Hyjek EM, et al (1998) Transformation of primary human endothelial cells by Kaposi's sarcoma-associated herpesvirus. Nature 394: 588–592. 9707121 10.1038/29093

[26] Cheng F, Pekkonen P, Laurinavicius S, Sugiyama N, Henderson S, et al (2011) KSHV-initiated notch activation leads to membrane-type-1 matrix metalloproteinase-dependent lymphatic endothelial-to-mesenchymal transition. Cell host & microbe 10: 577–590. 22177562 10.1016/j.chom.2011.10.011

[27] Wang HW, Trotter MW, Lagos D, Bourboulia D, Henderson S, et al (2004) Kaposi sarcoma herpesvirus-induced cellular reprogramming contributes to the lymphatic endothelial gene expression in Kaposi sarcoma. Nature genetics 36: 687–693. 15220918 10.1038/ng1384

[28] Bartley TD, Hunt RW, Welcher AA, Boyle WJ, Parker VP, et al (1994) B61 is a ligand for the ECK receptor protein-tyrosine kinase. Nature 368: 558–560. 8139691 10.1038/368558a0

[29] Annamalai B, Liu X, Gopal U, Isaacs JS (2009) Hsp90 is an essential regulator of EphA2 receptor stability and signaling: implications for cancer cell migration and metastasis. Mol Cancer Res 7: 1021–1032. 19567782 10.1158/1541-7786.MCR-08-0582 PMC3155284

[30] Kawabe M, Mandic M, Taylor JL, Vasquez CA, Wesa AK, et al (2009) Heat shock protein 90 inhibitor 17-dimethylaminoethylamino-17-demethoxygeldanamycin enhances EphA2+ tumor cell recognition by specific CD8+ T cells. Cancer Res 69: 6995–7003. 19690146 10.1158/0008-5472.CAN-08-4511 PMC2745213

[31] Kertesz N, Krasnoperov V, Reddy R, Leshanski L, Kumar SR, et al (2006) The soluble extracellular domain of EphB4 (sEphB4) antagonizes EphB4-EphrinB2 interaction, modulates angiogenesis, and inhibits tumor growth. Blood 107: 2330–2338. 16322467 10.1182/blood-2005-04-1655 PMC1895726

[32] Djokovic D, Trindade A, Gigante J, Badenes M, Silva L, et al (2010) Combination of Dll4/Notch and Ephrin-B2/EphB4 targeted therapy is highly effective in disrupting tumor angiogenesis. BMC cancer 10: 641. 21092311 10.1186/1471-2407-10-641 PMC3001720

[33] Spannuth WA, Mangala LS, Stone RL, Carroll AR, Nishimura M, et al (2010) Converging evidence for efficacy from parallel EphB4-targeted approaches in ovarian carcinoma. Molecular cancer therapeutics 9: 2377–2388. 20682653 10.1158/1535-7163.MCT-10-0200 PMC2933364

[34] Hahn AS, Kaufmann JK, Wies E, Naschberger E, Panteleev-Ivlev J, et al (2012) The ephrin receptor tyrosine kinase A2 is a cellular receptor for Kaposi's sarcoma-associated herpesvirus. Nature medicine 18: 961–6. 10.1038/nm.2805 PMC3645317 22635007

[35] Chakraborty S, Veettil MV, Bottero V, Chandran B (2012) Kaposi's sarcoma-associated herpesvirus interacts with EphrinA2 receptor to amplify signaling essential for productive infection. Proceedings of the National Academy of Sciences of the United States of America 109: E1163–1172. 22509030 10.1073/pnas.1119592109 PMC3358903

[36] Dittmer DP (2003) Transcription profile of Kaposi's sarcoma-associated herpesvirus in primary Kaposi's sarcoma lesions as determined by real-time PCR arrays. Cancer Research 63: 2010–2015. 12727810

[37] Dittmer DP (2011) Restricted Kaposi's sarcoma (KS) herpesvirus transcription in KS lesions from patients on successful antiretroviral therapy. mBio 2: e00138–00111. 22045987 10.1128/mBio.00138-11 PMC3202757

[38] Ballestas ME, Chatis PA, Kaye KM (1999) Efficient persistence of extrachromosomal KSHV DNA mediated by latency-associated nuclear antigen. Science 284: 641–644. 10213686 10.1126/science.284.5414.641

[39] Kaul R, Verma SC, Robertson ES (2007) Protein complexes associated with the Kaposi's sarcoma-associated herpesvirus-encoded LANA. Virology 364: 317–329. 17434559 10.1016/j.virol.2007.03.010 PMC4067005

[40] Chen W, Dittmer DP (2011) Ribosomal protein S6 interacts with the latency-associated nuclear antigen of Kaposi's sarcoma-associated herpesvirus. Journal of Virology 85: 9495–9505. 21734034 10.1128/JVI.02620-10 PMC3165752

[41] Si H, Verma SC, Robertson ES (2006) Proteomic analysis of the Kaposi's sarcoma-associated herpesvirus terminal repeat element binding proteins. J Virol 80: 9017–9030. 16940514 10.1128/JVI.00297-06 PMC1563930

[42] Barbera AJ, Chodaparambil JV, Kelley-Clarke B, Joukov V, Walter JC, et al (2006) The nucleosomal surface as a docking station for Kaposi's sarcoma herpesvirus LANA. Science 311: 856–861. 16469929 10.1126/science.1120541

[43] Shamay M, Liu J, Li R, Liao G, Shen L, et al (2012) A protein array screen for Kaposi's sarcoma-associated herpesvirus LANA interactors links LANA to TIP60, PP2A activity, and telomere shortening. Journal of Virology 86: 5179–5191. 22379092 10.1128/JVI.00169-12 PMC3347335

[44] An FQ, Compitello N, Horwitz E, Sramkoski M, Knudsen ES, et al (2005) The latency-associated nuclear antigen of Kaposi's sarcoma-associated herpesvirus modulates cellular gene expression and protects lymphoid cells from p16 INK4A-induced cell cycle arrest. The Journal of biological chemistry 280: 3862–3874. 15525642 10.1074/jbc.M407435200

[45] Fujimuro M, Wu FY, ApRhys C, Kajumbula H, Young DB, et al (2003) A novel viral mechanism for dysregulation of beta-catenin in Kaposi's sarcoma-associated herpesvirus latency. Nat Med 9: 300–306. 12592400 10.1038/nm829

[46] Friborg J Jr, Kong W, Hottiger MO, Nabel GJ (1999) p53 inhibition by the LANA protein of KSHV protects against cell death. Nature 402: 889–894. 10622254 10.1038/47266

[47] Grundhoff A, Ganem D (2004) Inefficient establishment of KSHV latency suggests an additional role for continued lytic replication in Kaposi sarcoma pathogenesis. The Journal of clinical investigation 113: 124–136. 14702116 10.1172/JCI200417803 PMC300762

[48] Chen W, Hilton IB, Staudt MR, Burd CE, Dittmer DP (2010) Distinct p53, p53:LANA, and LANA complexes in Kaposi's Sarcoma--associated Herpesvirus Lymphomas. Journal of Virology 84: 3898–3908. 20130056 10.1128/JVI.01321-09 PMC2849491

[49] Fujimuro M, Hayward SD (2003) The latency-associated nuclear antigen of Kaposi's sarcoma-associated herpesvirus manipulates the activity of glycogen synthase kinase-3beta. Journal of Virology 77: 8019–8030. 12829841 10.1128/JVI.77.14.8019-8030.2003 PMC161926

[50] Wen KW, Damania B (2010) Hsp90 and Hsp40/Erdj3 are required for the expression and anti-apoptotic function of KSHV K1. Oncogene 29: 3532–3544. 20418907 10.1038/onc.2010.124 PMC2908282

[51] Sin SH, Roy D, Wang L, Staudt MR, Fakhari FD, et al (2007) Rapamycin is efficacious against primary effusion lymphoma (PEL) cell lines in vivo by inhibiting autocrine signaling. Blood 109: 2165–2173. 17082322 10.1182/blood-2006-06-028092 PMC1801055

[52] Fakhari FD, Dittmer DP (2002) Charting latency transcripts in Kaposi's sarcoma-associated herpesvirus by whole-genome real-time quantitative PCR. Journal of Virology 76: 6213–6223. 12021355 10.1128/JVI.76.12.6213-6223.2002 PMC136228

[53] Staudt MR, Kanan Y, Jeong JH, Papin JF, Hines-Boykin R, et al (2004) The tumor microenvironment controls primary effusion lymphoma growth in vivo. Cancer Research 64: 4790–4799. 15256448 10.1158/0008-5472.CAN-03-3835

[54] Eccles SA, Massey A, Raynaud FI, Sharp SY, Box G, et al (2008) NVP-AUY922: a novel heat shock protein 90 inhibitor active against xenograft tumor growth, angiogenesis, and metastasis. Cancer Res 68: 2850–2860. 18413753 10.1158/0008-5472.CAN-07-5256

[55] Burch AD, Weller SK (2005) Herpes simplex virus type 1 DNA polymerase requires the mammalian chaperone hsp90 for proper localization to the nucleus. J Virol 79: 10740–10749. 16051866 10.1128/JVI.79.16.10740-10749.2005 PMC1182622

[56] Cerchietti LC, Lopes EC, Yang SN, Hatzi K, Bunting KL, et al (2009) A purine scaffold Hsp90 inhibitor destabilizes BCL-6 and has specific antitumor activity in BCL-6-dependent B cell lymphomas. Nat Med 15: 1369–1376. 19966776 10.1038/nm.2059 PMC2805915

[57] Pozo FM, Oda T, Sekimoto T, Murakumo Y, Masutani C, et al (2011) Molecular chaperone Hsp90 regulates REV1-mediated mutagenesis. Mol Cell Biol 31: 3396–3409. 21690293 10.1128/MCB.05117-11 PMC3147805

[58] Oda T, Hayano T, Miyaso H, Takahashi N, Yamashita T (2007) Hsp90 regulates the Fanconi anemia DNA damage response pathway. Blood 109: 5016–5026. 17327415 10.1182/blood-2006-08-038638

[59] Jensen MR, Schoepfer J, Radimerski T, Massey A, Guy CT, et al (2008) NVP-AUY922: a small molecule HSP90 inhibitor with potent antitumor activity in preclinical breast cancer models. Breast Cancer Res 10: R33. 18430202 10.1186/bcr1996 PMC2397535

[60] Stuhmer T, Zollinger A, Siegmund D, Chatterjee M, Grella E, et al (2008) Signalling profile and antitumour activity of the novel Hsp90 inhibitor NVP-AUY922 in multiple myeloma. Leukemia 22: 1604–1612. 18480838 10.1038/leu.2008.111

[61] Gaspar N, Sharp SY, Eccles SA, Gowan S, Popov S, et al (2010) Mechanistic evaluation of the novel HSP90 inhibitor NVP-AUY922 in adult and pediatric glioblastoma. Mol Cancer Ther 9: 1219–1233. 20457619 10.1158/1535-7163.MCT-09-0683 PMC2875164

[62] Stingl L, Stuhmer T, Chatterjee M, Jensen MR, Flentje M, et al (2010) Novel HSP90 inhibitors, NVP-AUY922 and NVP-BEP800, radiosensitise tumour cells through cell-cycle impairment, increased DNA damage and repair protraction. Br J Cancer 102: 1578–1591. 20502461 10.1038/sj.bjc.6605683 PMC2883148

[63] Ueno T, Tsukuda K, Toyooka S, Ando M, Takaoka M, et al (2011) Strong anti-tumor effect of NVP-AUY922, a novel Hsp90 inhibitor, on non-small cell lung cancer. Lung Cancer 76: 26–31. 21996088 10.1016/j.lungcan.2011.09.011

[64] Sarek G, Kurki S, Enback J, Iotzova G, Haas J, et al (2007) Reactivation of the p53 pathway as a treatment modality for KSHV-induced lymphomas. The Journal of clinical investigation 117: 1019–1028. 17364023 10.1172/JCI30945 PMC1810577

[65] Nomura N, Nomura M, Newcomb EW, Zagzag D (2007) Geldanamycin induces G2 arrest in U87MG glioblastoma cells through downregulation of Cdc2 and cyclin B1. Biochem Pharmacol 73: 1528–1536. 17324379 10.1016/j.bcp.2007.01.022

[66] Sun X, Barlow EA, Ma S, Hagemeier SR, Duellman SJ, et al (2010) Hsp90 inhibitors block outgrowth of EBV-infected malignant cells in vitro and in vivo through an EBNA1-dependent mechanism. Proc Natl Acad Sci U S A 107: 3146–3151. 20133771 10.1073/pnas.0910717107 PMC2840277

[67] Petre CE, Sin SH, Dittmer DP (2007) Functional p53 signaling in Kaposi's sarcoma-associated herpesvirus lymphomas: implications for therapy. Journal of Virology 81: 1912–1922. 17121789 10.1128/JVI.01757-06 PMC1797584

[68] Roy D, Sin SH, Damania B, Dittmer DP (2011) Tumor suppressor genes FHIT and WWOX are deleted in primary effusion lymphoma (PEL) cell lines. Blood 118: e32–39. 21685375 10.1182/blood-2010-12-323659 PMC3158728

[69] Bhatt AP, Bhende PM, Sin SH, Roy D, Dittmer DP, et al (2010) Dual inhibition of PI3K and mTOR inhibits autocrine and paracrine proliferative loops in PI3K/Akt/mTOR-addicted lymphomas. Blood 115: 4455–4463. 20299510 10.1182/blood-2009-10-251082 PMC2881502

[70] Caldas-Lopes E, Cerchietti L, Ahn JH, Clement CC, Robles AI, et al (2009) Hsp90 inhibitor PU-H71, a multimodal inhibitor of malignancy, induces complete responses in triple-negative breast cancer models. Proc Natl Acad Sci U S A 106: 8368–8373. 19416831 10.1073/pnas.0903392106 PMC2688867

[71] Annamalai B, Liu XG, Gopal U, Isaacs JS (2009) Hsp90 Is an Essential Regulator of EphA2 Receptor Stability and Signaling: Implications for Cancer Cell Migration and Metastasis. Molecular Cancer Research 7: 1021–1032. 19567782 10.1158/1541-7786.MCR-08-0582 PMC3155284

[72] Masood R, Xia GB, Smith DL, Scalia P, Still JG, et al (2005) Ephrin B2 expression in Kaposi sarcoma is induced by human herpesvirus type 8: phenotype switch from venous to arterial endothelium. Blood 105: 1310–1318. 15471957 10.1182/blood-2004-03-0933

[73] Massey AJ, Schoepfer J, Brough PA, Brueggen J, Chene P, et al (2010) Preclinical antitumor activity of the orally available heat shock protein 90 inhibitor NVP-BEP800. Mol Cancer Ther 9: 906–919. 20371713 10.1158/1535-7163.MCT-10-0055

[74] Roy D, Dittmer DP (2011) Phosphatase and tensin homolog on chromosome 10 is phosphorylated in primary effusion lymphoma and Kaposi's sarcoma. The American journal of pathology 179: 2108–2119. 21819957 10.1016/j.ajpath.2011.06.017 PMC3181371

[75] Wang L, Dittmer DP, Tomlinson CC, Fakhari FD, Damania B (2006) Immortalization of primary endothelial cells by the K1 protein of Kaposi's sarcoma-associated herpesvirus. Cancer Research 66: 3658–3666. 16585191 10.1158/0008-5472.CAN-05-3680

[76] Ballestas ME, Kaye KM (2001) Kaposi's sarcoma-associated herpesvirus latency-associated nuclear antigen 1 mediates episome persistence through cis-acting terminal repeat (TR) sequence and specifically binds TR DNA. J Virol 75: 3250–3258. 11238851 10.1128/JVI.75.7.3250-3258.2001 PMC114118

[77] Qin H, Zhai Z, Powell-Coffman JA (2006) The Caenorhabditis elegans AHR-1 transcription complex controls expression of soluble guanylate cyclase genes in the URX neurons and regulates aggregation behavior. Developmental biology 298: 606–615. 16919260 10.1016/j.ydbio.2006.07.017

[78] Gasc JM, Renoir JM, Faber LE, Delahaye F, Baulieu EE (1990) Nuclear localization of two steroid receptor-associated proteins, hsp90 and p59. Experimental cell research 186: 362–367. 2298246 10.1016/0014-4827(90)90317-4

[79] Kang KI, Meng X, Devin-Leclerc J, Bouhouche I, Chadli A, et al (1999) The molecular chaperone Hsp90 can negatively regulate the activity of a glucocorticosteroid-dependent promoter. Proceedings of the National Academy of Sciences of the United States of America 96: 1439–1444. 9990042 10.1073/pnas.96.4.1439 PMC15481

[80] Lin JJ, Hemenway CS (2010) Hsp90 directly modulates the spatial distribution of AF9/MLLT3 and affects target gene expression. The Journal of biological chemistry 285: 11966–11973. 20159978 10.1074/jbc.M110.101642 PMC2852934

[81] Solier S, Kohn KW, Scroggins B, Xu W, Trepel J, et al (2012) Feature Article: Heat shock protein 90alpha (HSP90alpha), a substrate and chaperone of DNA-PK necessary for the apoptotic response. Proceedings of the National Academy of Sciences of the United States of America 109: 12866–12872. 22753480 10.1073/pnas.1203617109 PMC3420188

[82] Stecklein SR, Kumaraswamy E, Behbod F, Wang W, Chaguturu V, et al (2012) BRCA1 and HSP90 cooperate in homologous and non-homologous DNA double-strand-break repair and G2/M checkpoint activation. Proceedings of the National Academy of Sciences of the United States of America 109: 13650–5. 22869732 10.1073/pnas.1203326109 PMC3427093

[83] Solit DB, Chiosis G (2008) Development and application of Hsp90 inhibitors. Drug discovery today 13: 38–43. 18190862 10.1016/j.drudis.2007.10.007

[84] Geller R, Taguwa S, Frydman J (2012) Broad action of Hsp90 as a host chaperone required for viral replication. Biochimica et biophysica acta 1823: 698–706. 22154817 10.1016/j.bbamcr.2011.11.007 PMC3339566

[85] Travers J, Sharp S, Workman P (2012) HSP90 inhibition: two-pronged exploitation of cancer dependencies. Drug discovery today 17: 242–252. 22245656 10.1016/j.drudis.2011.12.021

[86] Brough PA, Aherne W, Barril X, Borgognoni J, Boxall K, et al (2008) 4,5-diarylisoxazole Hsp90 chaperone inhibitors: potential therapeutic agents for the treatment of cancer. J Med Chem 51: 196–218. 18020435 10.1021/jm701018h

[87] Pasquale EB (2010) Eph receptors and ephrins in cancer: bidirectional signalling and beyond. Nat Rev Cancer 10: 165–180. 20179713 10.1038/nrc2806 PMC2921274

[88] Wykosky J, Debinski W (2008) The EphA2 receptor and ephrinA1 ligand in solid tumors: function and therapeutic targeting. Mol Cancer Res 6: 1795–1806. 19074825 10.1158/1541-7786.MCR-08-0244 PMC3690928

[89] Sivakumar R, Sharma-Walia N, Raghu H, Veettil MV, Sadagopan S, et al (2008) Kaposi's sarcoma-associated herpesvirus induces sustained levels of vascular endothelial growth factors A and C early during in vitro infection of human microvascular dermal endothelial cells: biological implications. Journal of Virology 82: 1759–1776. 18057235 10.1128/JVI.00873-07 PMC2258737

[90] Yamanda S, Ebihara S, Asada M, Okazaki T, Niu K, et al (2009) Role of ephrinB2 in nonproductive angiogenesis induced by Delta-like 4 blockade. Blood 113: 3631–3639. 19218547 10.1182/blood-2008-07-170381

[91] Si H, Verma SC, Robertson ES (2006) Proteomic analysis of the Kaposi's sarcoma-associated herpesvirus terminal repeat element binding proteins. Journal of Virology 80: 9017–9030. 16940514 10.1128/JVI.00297-06 PMC1563930

[92] Hu J, Liu E, Renne R (2009) Involvement of SSRP1 in latent replication of Kaposi's sarcoma-associated herpesvirus. Journal of Virology 83: 11051–11063. 19710137 10.1128/JVI.00907-09 PMC2772803

